# Evaluation of the Antimicrobial and Organic Dye Removal Properties of Silver-Incorporated ZIF‑L Metal–Organic Framework Nanoparticles

**DOI:** 10.1021/acsomega.4c11412

**Published:** 2025-05-23

**Authors:** Sreejith Govindan, Rudra Nath Ghosh, Chaithra, Vaidehi Basavakumar Roopa, Ann Treasa Joseph, Ashutosh Gupta, Sudheer Moorkoth, Pramod K. Namboothiri, Mathew Peter

**Affiliations:** † Department of Basic Medical Sciences, Manipal, 76793 Manipal Academy of Higher Education, Madhav Nagar, Manipal, Karnataka 576104, India; ‡ Department of Biomedical Engineering, Manipal Institute of Technology, Manipal, Manipal Academy of Higher Education, Manipal, Madhav Nagar, Manipal, Karnataka 576104, India; § Department of Emergency Medicine, Kasturba Medical College, Manipal Academy of Higher Education, Manipal, Madhav Nagar, Manipal, Karnataka 576104, India; ∥ Department of Pharmaceutical Quality Assurance, Manipal College of Pharmaceutical Sciences, Manipal Academy of Higher Education, Manipal, Madhav Nagar, Manipal, Karnataka 576104, India

## Abstract

Bacterial resistance to antimicrobial agents is a serious problem encountered all over the world. With the misuse of antimicrobial agents for the treatment of human and animal diseases, large quantities of these agents find their way into our environment, polluting water and soil. Further, with industrialization and urbanization, several organic dyes are used as colorants for products. The waste products from such manufacturing processes find their way into our environment, especially in the less developed world. There is an urgent need to remediate the effects of organic dyes and develop a new class of materials that can tackle the effects of antimicrobial resistance. In this regard, the research in this paper pertains to the development of zinc imidazolate metal–organic framework (ZIF-L) nanoparticles and evaluation of their antimicrobial effects on both standard and clinically isolated strains of Staphylococcus aureus (S. aureus), Escherichia coli (E. coli), and Pseudomonas aeruginosa (P. aeruginosa). Further, in this work, we evaluated the removal of organic dyes using ZIF-L and Ag/ZIF-L nanoparticles. The synthesized nanoparticles were characterized using Fourier transform infrared (FTIR) spectroscopy, X-ray diffraction (XRD), scanning electron microscopy (SEM), energy-dispersive X-ray spectroscopy (EDS), thermal gravimetric analysis (TGA), UV–visible absorption spectroscopy, and band-gap energy analysis. The results for the antibacterial study showed that Ag/ZIF-L nanoparticles were superior to ZIF-L against S. aureus and P. aeruginosa strains tested, where the minimum bactericidal concentration (MBC) of Ag/ZIF-L was 2.5 mg/mL, whereas the MBC of ZIF-L was more than 10 mg/mL. In the case of E. coli strains, ZIF/L showed a better MBC value of less than 1.25 mg/mL, whereas for Ag/ZIF-L it was more than 1.25 mg/mL. Further, Ag/ZIF-L nanoparticles coated on a face mask and cotton gauze demonstrated superior antimicrobial activity compared to uncoated gauze and mask, whereas the Ag/ZIF-L-coated mask and gauze showed the zone of inhibition (ZOI) for all of the bacteria with P. aeruginosa having a higher ZOI value than S. aureus and E. coli. For quantification of the removal of organic dyes, ZIF-L and Ag/ZIF-L nanoparticles showed complete methylene blue dye removal from the solution at 150 min in the presence of sunlight. Under dark conditions, only 83% dye was removed by Ag/ZIF-L and 65% by ZIF-L. The results show that ZIF-L metal–organic frameworks have the potential to be used for clinical and environmental remediation applications.

## Graphical abstract

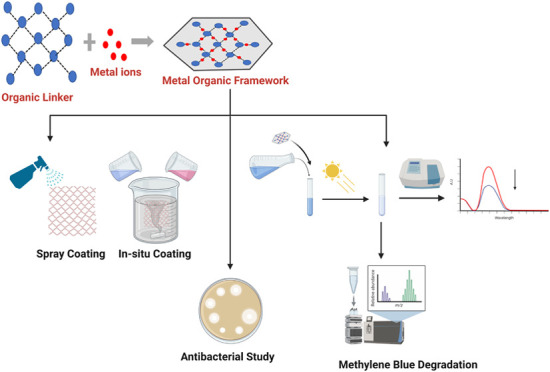

## 1 Introduction

The advent of industrialization and urbanization has caused the pollution of our environment.^1–3^ In both healthcare and animal husbandry, the rampant use of antimicrobial agents to tackle and prevent infections has led to the problem of antimicrobial resistance.^1,4–7^ Antimicrobial resistance is one significant new threat to our existence if we do not develop newer antimicrobial agents.^8^ The conventional antimicrobial agents used to prevent infection are based on organic molecules used in formulations to treat an infection. Modern animal husbandry practices utilize antibiotics to prevent infections and aid the growth of animals.^9–11^ The rampant use of antimicrobials in animals and humans has resulted in large doses of antimicrobials entering the environment and food chain.^9,12,13^ The pollution of the environment results in selection pressure in microbes, leading to favorable mutations in microbes.^14,15^ These mutations result in the development of antimicrobial resistance genes in microbes.^14,15^ Frequently, antimicrobial agents are used in subtherapeutic doses in animals as growth promoters.^16^ Subtherapeutic doses are perfect for the emergence of mutations that lead to the development of resistance genes. A growing concern is the resistance of microbes to medically relevant common antibiotic classes such as chloramphenicol, penicillin, tetracycline, sulfonamides, and fluoroquinolones attributed to their use in poultry.^17^ Food production in developing countries driven by market pressures and financial incentives continues to be the driving force for the rampant use of antimicrobial agents.

Another significant environmental problem is the growing issue of using organic dyes and compounds with rapid industrialization.^18^ Organic dyes and compounds are mainly used in pharmaceuticals, textiles, food, paper pulp, and cosmetic industries.^19^ The effluent from these industries contains a large amount of these dyes, leading to pollution of the environment.^20–23^ Most of these dyes are nontoxic and nondegradable, lingering in the environment for a long time. Dyes soluble in water are more difficult to remove from the waste and can be dispersed more widely in the environment.^24^ Most industrial dyes and their byproducts are toxic, mutagenic, or carcinogenic.^23,25,26^ Organic dyes entering the food chain can cause serious health issues in humans and animals.^26^ Oxidation, membrane filtration techniques, microbial technologies, bioelectrochemical degradation, and photocatalytic degradation are used to remove industrial dyes.^27^

Metal–organic frameworks (MOFs) are a class of porous materials prepared using metal and organic linkers.^28^ MOFs are three-dimensional structures with a very high surface area, which makes them ideal as absorbent materials.^29^ There has recently been tremendous interest in MOFs as adsorbent materials due to their porosity, simple synthesis route, stability, and low cost.^28^ MOFs are used for both liquid-phase and solid-phase extraction to remove organic pollutants from waste.^30^ MOFs are modified with other elements, extending their functionality as adsorbents for dye removal and degradation. Zeolitic imidazolate frameworks (ZIFs) are a class of MOFs that are widely studied and have applications in drug delivery, cancer therapeutics, environmental remediation, CO_2_ capture, and gas sensing.^31–34^ 2-Methylimidazole used for the preparation of MOFs has shown to enhance catalytic performance in reactions like Knoevenagel condensation.^35^ ZIF-8 is a zeolitic imidazolate framework prepared using a zinc metal precursor and an imidazole organic linker with a spherical morphology.^36^ ZIFs are porous tetrahedral-structured nanoparticles which are formed by the coordinate bond between Zn^2+^ ions and four imidazole linkers.^37^ ZIF-L is a leaf-shaped polymorph of ZIF-8 that can be transformed into ZIF-8 under certain conditions.^38^ The 2D structure of ZIF-L provides a greater surface area that can be beneficial in the catalytic reaction, where the Zn atoms are arranged in layers via π–π stacking, and the atoms at the edges of 2D ZIF-L have shown to have better catalytic activity.^39,40^ While ZIF-8 is widely studied as an absorbent material and an antimicrobial agent, there is limited literature on the use of ZIF-L for dye degradation and as an antimicrobial agent.

Recently, studies have shown the contact-killing properties of ZIF-L, making it an exciting material for coating surfaces to kill microbes.^41^ In this study, we prepared ZIF-L or silver-incorporated ZIF-L (Ag/ZIF-L) nanoparticles using a simple synthesis route using water as the solvent. Silver ions are known for their antimicrobial properties, which can be beneficial in applications where the MOF needs to prevent microbial growth. Further, silver ions and silver nanoparticles act as good catalytic agents by removing organic compounds from wastewater through photocatalytic reactions.^42^ Studies have shown that ZIF-based MOFs possess excellent photocatalytic degradation of organic dyes. A study by Li et al. showed the photodegradation properties of ZIF-67 MOFs doped with titanium dioxide (TiO_2_) nanoparticles for the degradation of dyes such as acid fuchsin, Congo red, erythrosine B, and Rose Bengal under light conditions.^43^ In another study conducted by Fan et al., silver-doped ZIF-8 nanoparticles were fabricated and were shown to be effective in removing organic dyes such as methylene blue from wastewater by photocatalytic degradation under UV light.^44^ To investigate these properties, we studied the dye removal properties of ZIF-L and Ag/ZIF-L under dark and sunlight conditions. Further, we studied the antimicrobial properties of the two compositions of ZIF-L. Finally, we coated ZIF-L and Ag/ZIF-L on bandages and masks and further demonstrated their ability to kill microbes.

## 2 Materials and Methods

### 2.1 Materials

Zinc nitrate hexahydrate (Zn­(NO_3_)_2_·6H_2_O) and 2-methylimidazole were purchased from Sigma-Aldrich. Silver nitrate was purchased from Loba Chemical (India). Methylene blue dye was purchased from SRL Chemicals. Muller Hinton broth, Muller Hinton agar plates, and sheep blood agar plates were purchased from HiMedia India.

### 2.2 Synthesis of ZIF-L- and ZIF-L-Doped Nanoparticles

ZIF-L nanoparticles were synthesized with modification of the previously described method by the precipitation reaction.^41^ Briefly, 0.04 M zinc nitrate solution was reacted with 0.03 M 2-methylimidazole solution (Figure 1), and the mixture was kept under constant stirring for 24 h at room temperature under dark conditions. The precipitate was then allowed to settle down, filtered, and collected. The product was then washed with 80% methanol and dried. The same procedure was followed for the preparation of Ag-doped ZIF (Ag/ZIF-L) nanoparticles, where 0.022 M silver nitrate was dissolved in the reaction mixture, followed by precipitation, filtration, and drying.

**Figure 1:**
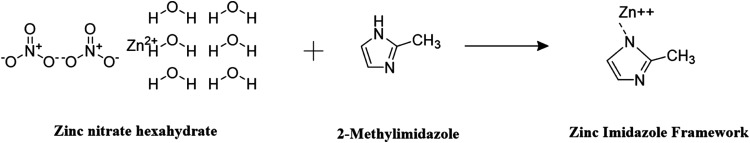
Preparation of ZIF-L nanoparticles and Ag/ZIF-L nanoparticles.

### 2.3 Characterization of ZIF-L and Ag/ZIF-L nanoparticles

#### 2.3.1 Fourier Transform Infrared Spectroscopy

Fourier transform infrared (FTIR) spectra of ZIF-L and Ag/ZIF-L were recorded using a Shimadzu FTIR spectrometer. 2 mg of sample was mixed with KBR and pelleted. The spectrum of the pellet was recorded from 4000 to 400 cm^–1^.

#### 2.3.2 X-ray Diffraction

X-ray diffraction (XRD) spectra of samples were recorded using a Rigaku MiniFlex diffractometer (Cu Kα radiation) operating at room temperature. The XRD reflections from 5 to 80° were recorded with a scan step size of 0.02 and a scan step time of 0.05 s.

#### 2.3.3 Scanning Electron Microscopy

A scanning electron microscope was used to image the samples to understand the size and morphology of the ZIF-L and Ag/ZIF-L nanoparticles. The samples were sputter-coated with gold and imaged by using a ZEISS EVO MA18. The samples’ energy-dispersive X-ray spectroscopy (EDS) spectra were recorded using an Oxford EDS system (X-Act) attached to a scanning electron microscope.

#### 2.3.4 Transmission Electron Microscopy

High-resolution transmission electron microscopy (HR-TEM; Talos F200X G2, Thermo Fisher) was used to determine the crystalline structure of Ag/ZIF-L and the presence of Ag nanoparticles on the ZIF-L structure. Selected area electron diffraction (SAED) was used to determine the crystal lattices of the Ag/ZIF-L nanoparticles, and energy-dispersive X-ray spectroscopy (EDS) was used for elemental mapping.

#### 2.3.5 Thermogravimetric Analysis

The thermal stability of nanoparticles was analyzed using a Hitachi STA 7200 thermogravimetric analyzer in a temperature range of 0–800 °C under a nitrogen atmosphere with a heating rate of 10 °C per minute.

#### 2.3.6 Surface Area Analysis

The Brunauer–Emmett–Teller (BET) surface area analysis was performed using nitrogen gas at a regeneration temperature of 100 °C using a BELSORP MINI X (Japan) instrument. The surface area and pore volume were calculated using the standard adsorption isotherm using BET, BJH, and Langmuir plotting methods.

#### 2.3.7 Inductively Coupled Plasma–Optical Emission Spectrometry

The amount of zinc and silver ions present in the nanoparticles was calculated by inductively coupled plasma–optical emission spectrometry (ICP–OES) analysis using a Thermo Fisher (iCAP PRO ICP–OES). For accurate reading in ppm, the nanoparticles were dissolved in acetic acid solution and then diluted 27-fold to avoid overflow of the data. 10 mg of nanoparticles was dissolved in acetic acid and diluted 27-fold. The amounts of zinc and silver ions present were determined using eq 1:

1 $$\mathrm{concentration}\;\mathrm{of}\;\mathrm{ions}\;\left(\mathrm{in}\;\mu g/\mathrm{mg}\;\mathrm{of}\;\mathrm{sample}\right)=\frac{\mathrm{dilution}\;\mathrm{factor}\times \mathrm{concentration}\;\left(\mathrm{in}\;\mathrm{ppm}\right)}{1000}$$

#### 2.3.8 UV–Vis Spectrometry and Band-Gap Analysis

Absorbance spectra of ZIF-L and Ag/ZIF-L were obtained using a Shimadzu UV-1900, Japan, at 190–300 nm wavelength. The Tauc plot was used to derive the band-gap energies of ZIF-L and Ag/ZIF-L nanoparticles.

### 2.4 Antimicrobial Properties of ZIF-L and Ag/ZIF-L Nanoparticles

The antimicrobial properties of ZIF-L and Ag/ZIF-L nanoparticles were analyzed using clinical strains and standards of Staphylococcus aureus (ATCC 25923), Pseudomonas aeruginosa (ATCC 27853), and Escherichia coli (ATCC 25922). Institutional ethics committee (IEC) approval (IEC No: IEC 846–2021) was obtained prior to the initiation of the antimicrobial study. The IEC exempted this study from obtaining informed consent from the patients as we used only the bacterial isolates obtained from the clinical specimens of anonymized patients. The antimicrobial properties were estimated by using minimum inhibitory concentration (MIC) and minimum bactericidal concentration (MBC). MBC was investigated to determine the lowest concentration of nanoparticles at which growth of 99.9% of bacterial population is inhibited. At first, the standard and clinical strains of bacteria were inoculated in Mueller-Hinton (MH) broth containing serially diluted nanoformulations (10, 5, 2.5, 1.25, 0.625, and 0.3 mg/mL concentration) of ZIF-L and Ag/ZIF-L nanoparticles and incubated in a shaking incubator at 37 °C for 6 h. The bacterial broth was then streaked onto the blood agar plate and incubated overnight at 37 °C. The least diluted MOF concentration, where zero bacterial growth is observed, was treated as the minimum concentration for total bactericidal activity. MIC was studied by preparing a solution of ZIF-L and silver-doped ZIF-L nanoparticles (Ag/ZIF-L) as well as iodine-doped ZIF-L (ZIF-I) and mannose-coated ZIF-L, Ag/ZIF-L, and ZIF-I nanoparticles (the preparation method is reported in the Supporting Information), which were added in MH broth at 10 mg/mL concentration. The standard and clinical strains of bacteria were plated in an MH agar plate, and 500 μL of the nanoparticle solution was added to a punch well having a diameter of 8 mm and a volume of 550–600 mm^3^, which was made in the plate using a biopsy puncher. After overnight incubation at 37 °C, the zones of inhibition for both ZIF and ZIF-doped nanoparticles were calculated. Similarly, the MIC of in-situ-coated, spray-coated, and noncoated gauze and mask was determined. The details of methodology of the coating process are provided in the Supporting Information. Ag/ZIF-L nanoparticles at a concentration of 10 mg/mL were used for studying the antimicrobial properties of coated gauze and mask.

### 2.5 Dye Absorption Studies of Nanoparticles

Methylene blue was used as the model dye to study dye removal using prepared ZIF-L and Ag/ZIF-L nanoparticles. The dye was dissolved in 5 mL of deionized water at 10, 100, and 1000 μM concentrations, and ZIF-L and Ag/ZIF-L nanoparticles (40 mg) were added to the dye. We exposed the dye incubated with nanoparticles to bright sunlight to study the photocatalytic activity. For comparison, we also kept the dye with nanoparticles and recorded the removal of the dye from the solution under dark conditions. Methylene blue dye adsorbed by the ZIF-L and Ag/ZIF-L nanoparticles under light and dark conditions was evaluated using UV–vis spectrophotometry, where after a certain period of time, the solvent was taken to take the spectral scan reading for the test and control groups (dye without nanoparticles). The ZIF-L and Ag/ZIF-L nanoparticles after methylene blue dye removal were characterized using FTIR spectroscopy (Jasco ATR-FTIR, Japan). The study was repeated with other complex azo dyes like Congo red, and the photocatalytic activity of both ZIF-L and Ag/ZIF-L under light and dark conditions was determined.

### 2.6 Analysis of Methylene Blue Dye Degradation Using LC–MS

The degradation byproducts of methylene blue dye were analyzed using a Dionex Ultimate 3000 liquid chromatograph coupled to an LTQ XL mass spectrometer (Thermo Scientific). ZIF-L and Ag/ZIF-L nanoparticles were added to methylene blue dye at 10 mg/mL concentration and analyzed under light (sunlight) and dark conditions for their photocatalytic activity. The methylene blue byproduct was separated using a Genesis C18 (100 × 4.6 mm) 4 μm particle size column, and the mass detection of the byproducts was performed using an ion-trap LTQ XL mass spectrometer. The mobile phase used for the study consisted of a combination of two solutions (0.1% formic acid (30%)/methanol (70%)), and a volume of 2 μL was injected into the column at a flow rate of 0.5 mL/min. For mass analysis, the ESI conditions for ion-trap mass spectroscopy were as follows: heater temperature: 102.81 °C; sheath gas flow rate: 35 (arbitrary units); auxiliary gas flow rate: 10 (arbitrary units); spray voltage: 4.00 kV; spray current: 0.08 μA; capillary temperature: 250.00 °C; capillary voltage: 30.70 V. The mass range was selected from *m*/*z* 110 to *m*/*z* 400, and the software used for analysis was Chromeleon 7.3.2 CDS (Thermo Fisher).

## 3 Results and Discussion

### 3.1 Characterization of ZIF-L and Ag/ZIF-L

#### 3.1.1 FTIR Analysis

The results of FTIR studies are shown in Figure 2A. Characteristic peaks were found at 1594 cm^–1^ (C=N stretching vibration), 1302, 1154, and 993 cm^–1^ (C–N bending vibration), and 753 and 681 cm^–1^ (C–H bending vibration) for 2-methylimidazole, ZIF-L, and Ag/ZIF-L. A distinct peak at 421 cm^–1^ attributed to the vibrations of Zn–N was observed in the ZIF-L and Ag/ZIF-L groups, which is absent in 2-methylimidazole. The presence of these vibrational peaks confirms the successful synthesis of the zinc imidazolate framework. We did not observe any additional peaks due to doping with silver. These results agree with the reported literature.^45,46^

**Figure 2:**
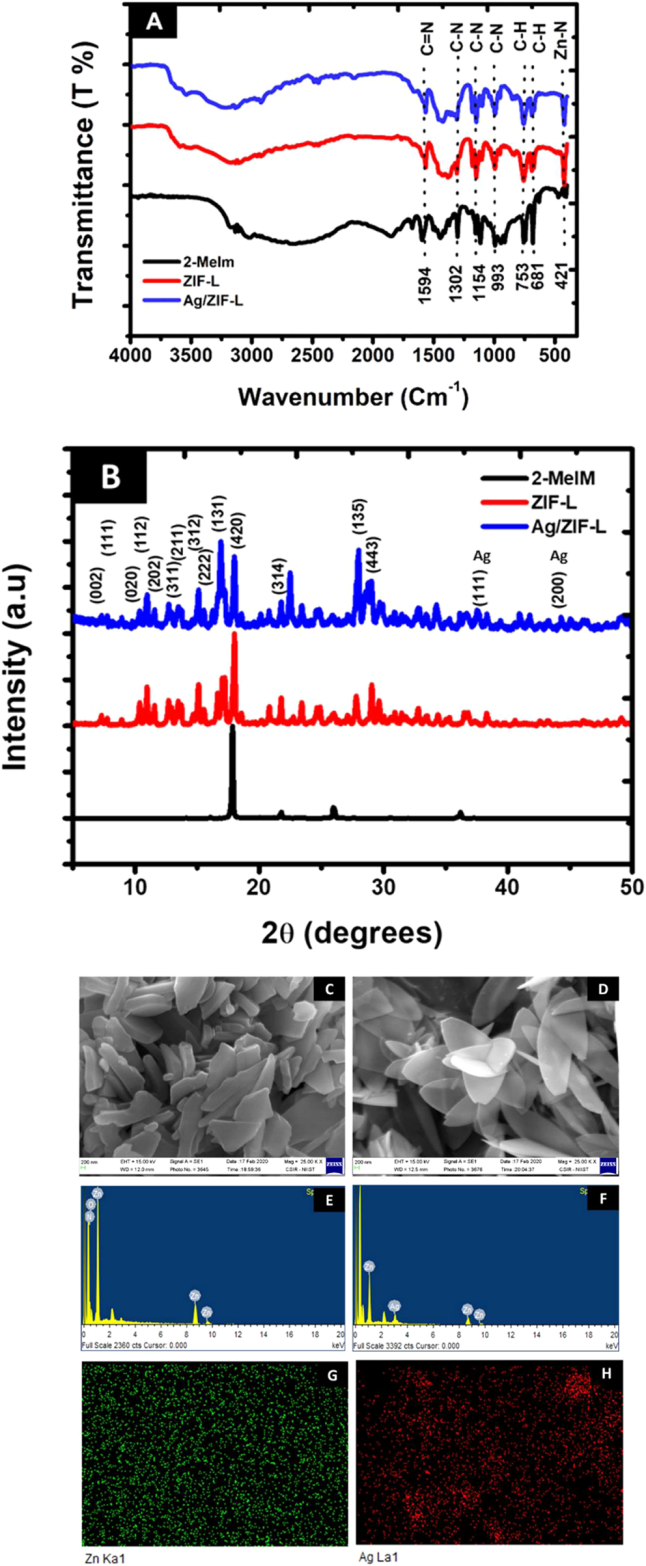
Characterization of the prepared ZIF-L nanoparticles. (A) FTIR spectra of prepared ZIF-L particles (2-MeIm, 2-methylimidazole, ZIF-L, and Ag/ZIF-L-undoped and silver-doped ZIF-L). (B) X-ray diffraction patterns of prepared nanoparticles (2-MeIM, 2-methylimidazole, ZIF-L, and Ag/ZIF-L undoped and silver-doped ZIF-L). (C and D) Scanning electron micrographs of ZIF-L and Ag/ZIF-L nanoparticles. (E and F) Energy-dispersive spectra of ZIF-L and Ag/ZIF-L nanoparticles. (G and H) Energy-dispersive spectral maps of Ag/ZIF-L nanoparticles.

#### 3.1.2 Powder X-ray Diffraction Studies

Figure 2B shows the X-ray diffraction patterns of 2-methylimidazole, ZIF-L, and Ag/ZIF-L. The major peaks for ZIF-L and Ag/ZIF-L were observed around 2θ angles of 7.3 (200), 7.4 (111), 10.4 (020), 10.9 (112), 11.5 (202), 12.6 (220), 12.9 (311), 13.5 (311), 15.1 (312), 15.6 (222), 17.0 (023), 17.2 (402), 18.0 (420), 21.7 (314), 27.7 (135), and 29.0° (443).^47^ The peaks at 37.6 (111) and 44.2° (200) are attributed to reflections from planes from Ag/ZIF-L, which are missing in ZIF-L particles.^48^ These peaks are attributed to reflection from the silver. These results confirm previous reports and the formation of ZIF-L and Ag/ZIF-L particles.

#### 3.1.3 Scanning Electron Microscopy and Energy-Dispersive Spectroscopy

SEM and EDS analyses were performed to determine the morphology and elemental composition. Figure 2C,D shows the scanning electron micrographs of the prepared ZIF-L and Ag/ZIF-L nanoparticles. We can observe the two-dimensional nature of prepared nanoparticles from SEM micrographs. The morphology of ZIF-L nanoparticles was rod-shaped, while that of Ag/ZIF-L was more leaf-like 2D crystals, in good agreement with the reported literature.^38^ The size of the particles is less than 200 nm in thickness and micrometer scale in length and width. Figure 2E,F shows the EDS spectra of the prepared ZIF and Ag/ZIF-L nanoparticles. The spectra for ZIF-L show the presence of zinc and nitrogen atoms, while the spectra for Ag/ZIF-L additionally show the presence of silver, confirming the incorporation of silver. Figure 2G,H represents the EDS maps of Ag/ZIF-L, and the maps show the uniform distribution of zinc and silver within the ZIF-L nanoparticles. These results further confirm the presence of silver in the doped ZIF-L sample’s successful preparation of these particles.

#### 3.1.4 Transmission Electron Microscopy and Energy-Dispersive Spectroscopy

To determine the presence of Ag nanoparticles on Ag/ZIF-L MOFs, transmission electron microscopy was performed. The TEM images show a flower-shaped leaflet structure of Ag/ZIF-L, which correlates with the SEM images. The presence of Ag nanoparticles is seen as dark spots on the ZIF-L MOFs, as illustrated by the TEM image in Figure 3A and STEM image in Figure 3B, and this is confirmed by EDS mapping and elemental analysis, as illustrated in Figure 3C. The crystalline structure of Ag/ZIF-L MOFs was determined using selected area electron diffraction (SAED) analysis, and the result is illustrated in Figure 3D, and the *d*-spacing of Ag atoms in the Ag/ZIF-L nanoparticles was calculated to be 0.231 nm, as illustrated in Figure 3E, which is the same as the *d*-spacing of silver atoms in X-ray diffraction patterns of silver nanoparticles corresponding to the (111) plane.

**Figure 3:**
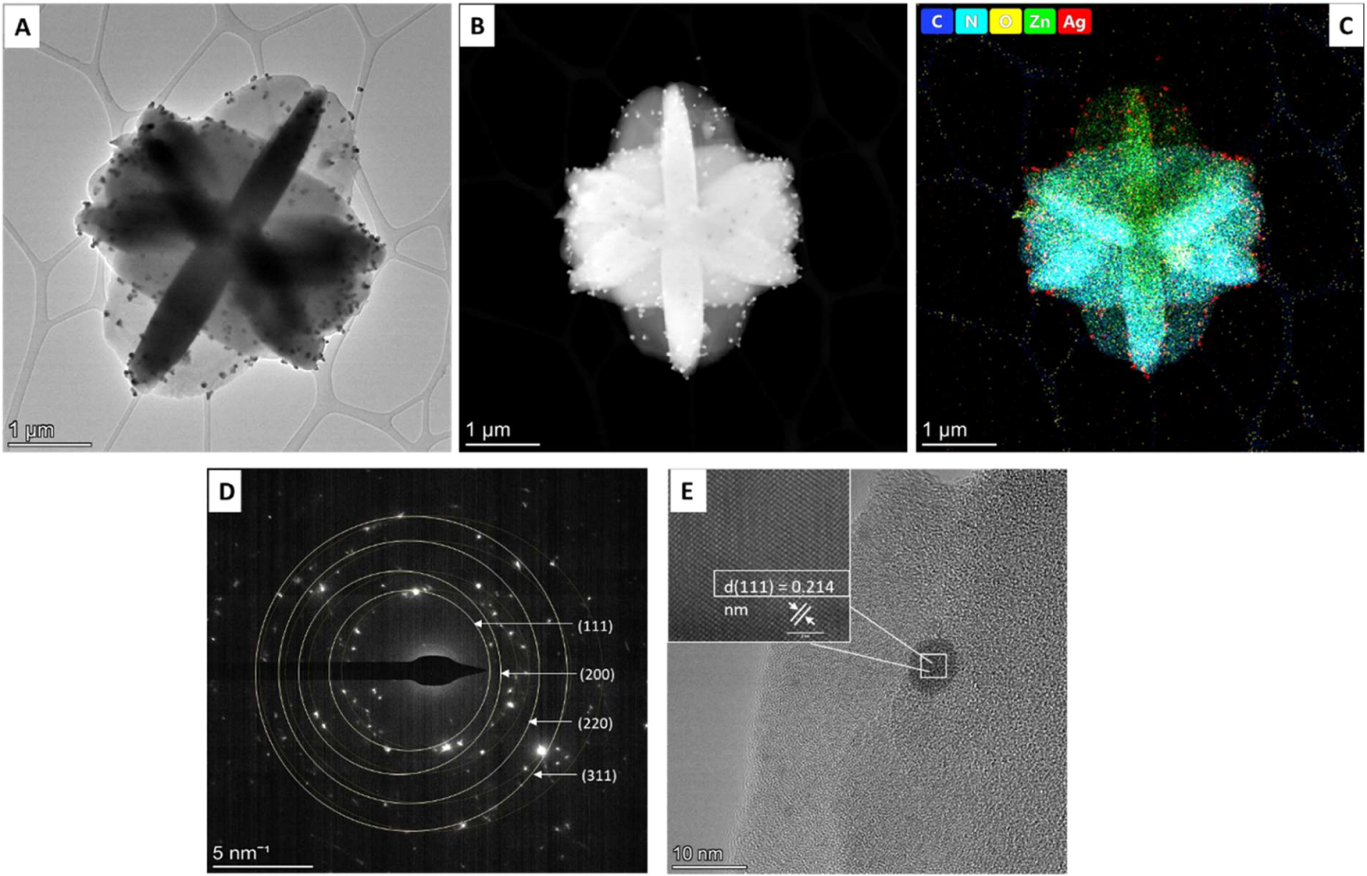
Transmission electron microscopic (TEM) analysis of Ag/ZIF-L nanoparticles. (A) TEM image of Ag/ZIF-L nanoparticles. (B) STEM image of Ag/ZIF-L nanoparticles. (C) Energy-dispersive spectrum and elemental mapping (carbon, nitrogen, oxygen, zinc, and silver) of Ag/ZIF-L nanoparticles. (D) Selected area electron diffraction (SAED) image of Ag/ZIF-L nanoparticles. (E) *d*-spacing of Ag atoms in Ag/ZIF-L nanoparticles.

#### 3.1.5 Thermogravimetric Analysis

The thermal analysis of prepared nanoparticles was performed to understand the thermal stability of the nanoparticles. Figure 4A shows the thermogravimetric curves of ZIF-L and Ag/ZIF-L under a nitrogen atmosphere, while Figure 4B shows the derivative thermogravimetric plot of prepared nanoparticles. TGA of ZIF-L nanoparticles showed a 6.48% weight loss between 100 and 120 °C, which could be attributed to the loss of water molecules. The second weight loss of around 12.96% was observed between 220 and 320 °C, corresponding to the removal of moisture or residual ligand from synthesis. The network structure of ZIF-L collapses between 400 and 550 °C. This is indicated by a significant weight loss of 38% in this range. We observed 24.8% of residue between 500 and 800 °C for the ZIF-L group. In contrast, Ag/ZIF-L showed 5% weight loss between 100 and 220 °C and 21% in the 220–320 °C range. This can be attributed to the lower adsorption of water on Ag/ZIF-L compared to ZIF-L due to silver occupying the pores in Ag/ZIF-L. However, between 220 and 320 °C, residual water and ligands may degrade or desorb, resulting in weight loss of the sample. From the DTG trace (Figure 3B), we observed a change in the DTG peak in the range of 220–320 °C. Ag/ZIF showed a maximum rate of weight loss at 286 °C in comparison to ZIF-L samples. Ag/ZIF-L showed a 28.54% weight loss between 400 and 550 °C and 32.1% residue remaining compared to ZIF-L. The higher residue in Ag/ZIF-L (32.1%) compared to ZIF-L (24.8%) can be attributed to silver within the ZIF-L network.

**Figure 4:**
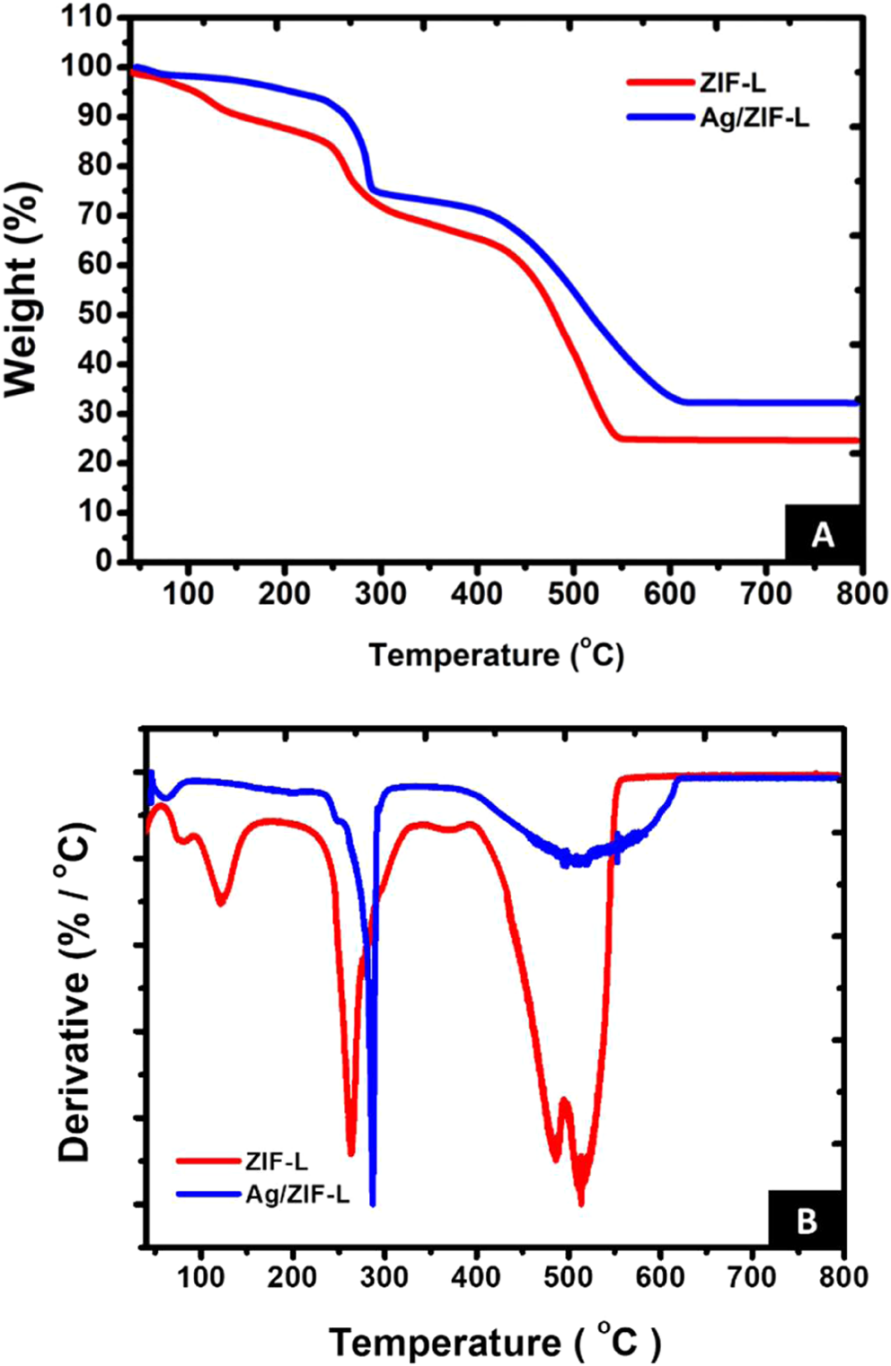
Thermal analysis of ZIF-L nanoparticles. (A) Thermogravimetric analysis of ZIF-L and Ag/ZIF-L under a nitrogen atmosphere. (B) Derivative thermogravimetric plots of ZIF-L and Ag/ZIF-L nanoparticles.

#### 3.1.6 BET Analysis and ICP–OES Analysis

The surface area and pore volume of ZIF-L and Ag/ZIF-L were determined using BET, BJH, and Langmuir plotting methods, and the results are shown in Table 1. The surface areas of ZIF-L and Ag/ZIF-L particles measured in our study at 100 °C are similar to the results reported by others in the literature.^38,49^ The surface area and pore volume recorded were higher for the Ag/ZIF-L group than for the ZIF-L group. Although, in the literature, it has been reported that doping of Ag with ZIF-8 nanoparticles did not have any significant change in the surface area or pore volume,^50^ our study with Ag-doped ZIF-L nanoparticles has shown a significant increase in the surface area, which may be due to the Ag nanoparticle dispersion on the surface of the ZIF-L nanoparticles, leading to the formation of additional micropores and mesopores, which increase the overall surface area.

**Table 1:** Surface Area and Pore Volume Analyses of ZIF-L and Ag/ZIF-L Nanoparticles Using the BET and BJH Methods

	BET plot		BJH plot		Langmuir plot
	surface area (m^2^/g)	total pore volume (cm^3^/g)	surface area (m^2^/g)	mesoporous volume (cm^3^/g)	Langmuir surface area (m^2^/g)
ZIF-L	2.6402	0.0133	2.4286	0.0196	9.9107
Ag/ZIF-L	6.7085	0.0245	10.327	0.0206	29.208

To determine the amount of silver and zinc incorporated within the particles, ZIF-L or Ag/ZIF-L nanoparticles were acid-digested, and the resultant concentration of the digested solution was determined using ICP–OES. The results show that zinc concentration ranged from about 15 to 17 μg per mg ZIF-L or Ag/ZIF-L nanoparticles. Similarly, the silver concentration was determined to be 1.8–1.95 μg per mg Ag/ZIF-L nanoparticles, as reported in Table 2. The concentration of zinc was calculated using the empirical formula weight for ZIF-L (Zn­(mim)­2·(Hmim)­1/2·(H2O)­3/2), which was found to be around 295, which corresponds to around 22% of zinc by weight.^51^ We found a good arrangement between the concentrations of zinc ions, as determined by our potentiometric titration (205 μg/mg ZIF-L) and the empirically calculated amount per milligram of nanoparticles. Further, from residual mass data using TGA, we found that the silver amount was around 7% by weight. This is again in good agreement with the potentiometric titration data for the weight of silver per milligram ZIF-L. The silver ion concentration calculated by the potentiometric method was around 100 μg/mg Ag/ZIF-L.

**Table 2:** Zinc and Silver Ion Concentrations Present in ZIF-L and Ag/ZIF-L Nanoparticles

	concentration of Zn ion (μg/mg)	concentration of Ag ion (μg/mg)
ZIF-L	16.93 ± 0.96	
Ag/ZIF-L	16.43 ± 93	1.89 ± 0.6

#### 3.1.7 UV–Visible Absorption Spectroscopy and Band-Gap Analysis

The UV–visible absorbance spectra for ZIF-L and ZIF-Ag show absorption peaks of ZIF-L at 206 nm and Ag/ZIF-L at 195 nm, as illustrated in Figure 5A. The absorption peak of ZIF-L at 206 nm is similar to that reported in the literature, where the absorption peak of ZIF-L is around 210 nm.^52^ The band-gap analysis of ZIF-L and Ag/ZIF-L was performed using the Tauc plot method. The band gap of ZIF-L was calculated to be around 5.35 eV, slightly higher than that in the previous literature, which is around 4.8–5.0 eV for ZIF-L.^52,53^ The band-gap energy of Ag/ZIF-L was 5.582 eV, as illustrated in Figure 5B. The shift in the band gap of Ag/ZIF-L may be attributed to the strain developed in the ZIF-L structure due to the presence of silver nanoparticles, leading to band-gap widening.^54^

**Figure 5:**
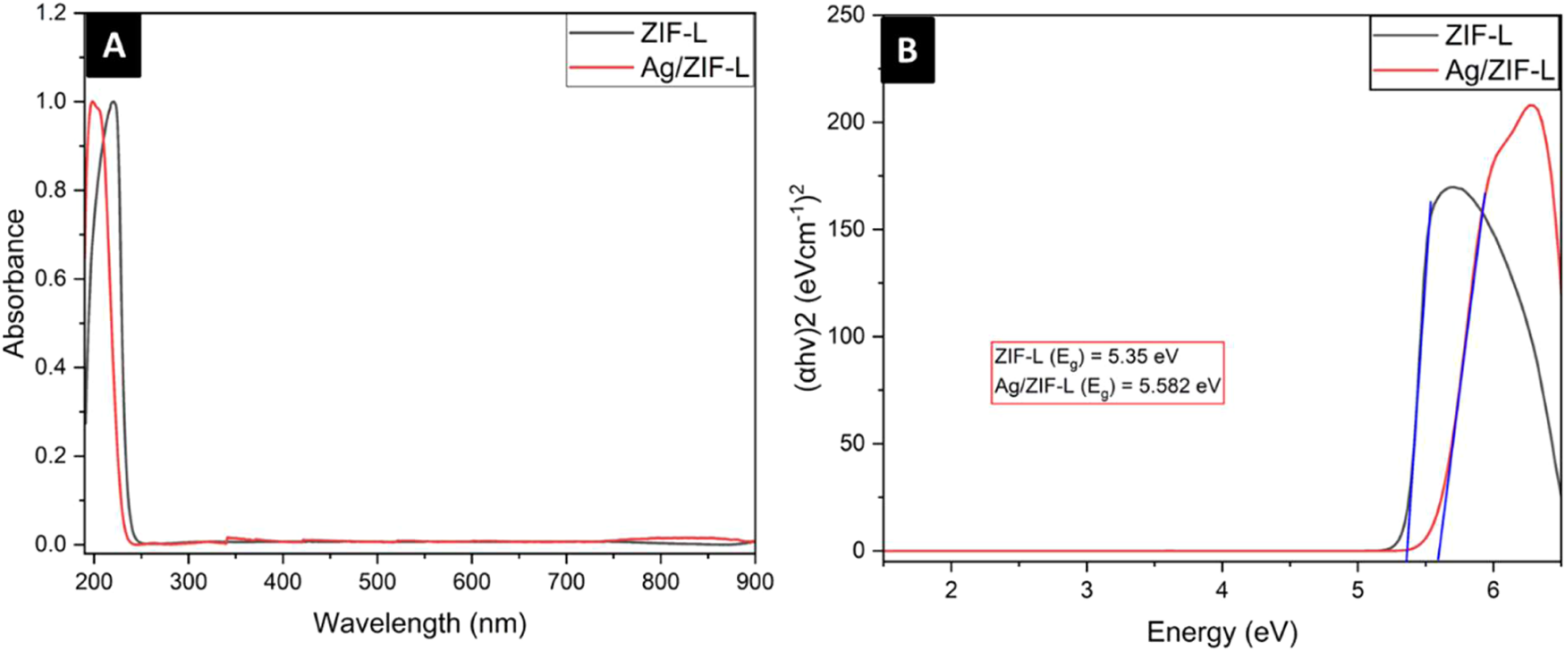
(A) UV–visible spectra of ZIF-L and Ag/ZIF-L nanoparticles. (B) Band-gap energy determination using the Tauc plot.

### 3.2 Antimicrobial Properties of ZIF-L and Ag/ZIF-L

The antimicrobial properties of ZIF-L and Ag/ZIF-L nanoparticles were tested on clinical isolates and standard ATCC strains of S. aureus, E. coli, and P. aeruginosa bacteria. We also prepared iodine-loaded I/ZIF-L and mannose-coated ZIF-L, Ag/ZIF-L, and I/ZIF-L. Mannose is reported to show antimicrobial effects.^55^ Figure 6 shows the punch well method with nanoparticles at 10 mg/mL concentration to study the zone of inhibition from the respective nanoformulation with the bacterial strains. We observed the most significant activity in the Ag/ZIF-L group compared to ZIF-L and I/ZIF-L groups. The addition of mannose or iodine did show any significant antimicrobial activity. Figure 7 shows the minimum bactericidal concentration (MBC) for ZIF-L and Ag/ZIF-L against respective strains for 10, 5, 2.5, 1.35, 0.625, and 0.3 mg/mL concentrations of nanoparticles. The bactericidal activities of different silver-based MOFs corresponding to the zone of inhibition (ZOI) are presented in Table 3. The results from Table 4 show the minimum bactericidal concentration (MBC) for various concentrations of nanoparticles. We found that ZIF-L nanoparticles required a higher dose for killing the clinical strain (2.5 mg/mL) compared to the standard strain (1.25 mg/mL) for E. coli. In the case of S. aureus, ZIF-L was only active against the clinical strain (5 mg/mL) and needed a higher concentration than the tested sample (>10 mg/mL) to kill the standard strain. In the case of P. aeruginosa, ZIF-L required a higher concentration (>10 mg/mL) than the tested sample to inhibit the bacteria for both strains. In the case of Ag/ZIF-L, a similar concentration (2.5 mg/mL) to ZIF-L was required for inhibiting the E. coli clinical strain. However, a higher concentration was required for Ag/ZIF-L (5 mg/mL) compared to ZIF-L (1.25 mg/mL) for the standard strain. Ag/ZIF-L showed inhibition of S. aureus at 1.25 mg/mL concentration compared to >10 mg/mL for standard strain and lower concentration (1.25 mg/mL) for clinical strains compared to ZIF-L. Ag/ZIF-L (0.625 or 1.25 mg/mL) was more potent in inhibiting P. aeruginosa compared to ZIF-L (>10 mg/mL). These studies demonstrate the incorporation of silver to aid and be beneficial for inhibiting bacteria. The mechanism of action by which silver or silver nanoparticles help in killing bacteria is through binding to the bacterial cell wall and subsequently disrupting the cell membrane permeability. It has been reported that silver ions inhibit many enzymes and their pathways such as respiratory enzyme dehydrogenases by binding to the thiol groups of the enzyme. Silver can also bind to the phosphorus and sulfur moieties of DNA and alter the replication process that can lead to the killing of the bacteria. Moreover, the accumulation of silver in the bacterial cytoplasm can lead to the binding of silver ions to ribosomal proteins, thus disrupting the protein synthesis process. It is also shown to increase ROS production in cells leading to cell death.^56–58^ To replicate the antimicrobial properties of Ag/ZIF-L nanoparticles on coated surfaces against common pathogens, we spray-coated Ag/ZIF-L nanoparticles on the surface of a surgical mask or cotton gauze. On cotton gauze, we further coated Ag/ZIF-L by in situ growth of particles on the surface of cotton gauze by immersing the gauze in the reaction solution during the synthesis process. The SEM images and EDS mapping of the coated cotton gauze and mask with ZIF-L and Ag/ZIF-L are shown in Supporting Information Figure S1. Figure 8A–F shows the antimicrobial properties of coated and uncoated samples against the strains of bacteria. It can be observed that coated mask/gauze (samples 2, 3, and 4) shows inhibition of bacterial growth for both standard and clinical strains in comparison to uncoated control samples (samples 1 and 5). We observed slightly higher activity against P. aeruginosa compared to E. coli and S. aureus. However, these results were not significant. From Figure 8, we can observe the apparent difference in the zone of inhibition between control samples (noncoated) and coated samples in clinical (Figure 8G) and standard strains (Figure 8H). Our studies are similar to other reported literature of ZIF-8 doped with other metal ions, where the MIC values for standard strains of E. coli, P. aeruginosa, and S. aureus were determined as 0.5, 0.25, and 1.0 mg/mL, respectively.^59,60^ However, our studies highlight the antimicrobial activity of clinically isolated strains of bacterial samples, which require either lower or higher doses than standard strains. The coating and antimicrobial activity of ZIF-L-coated gauze mask also agree with similar work reported in the literature.^61^

**Figure 6:**
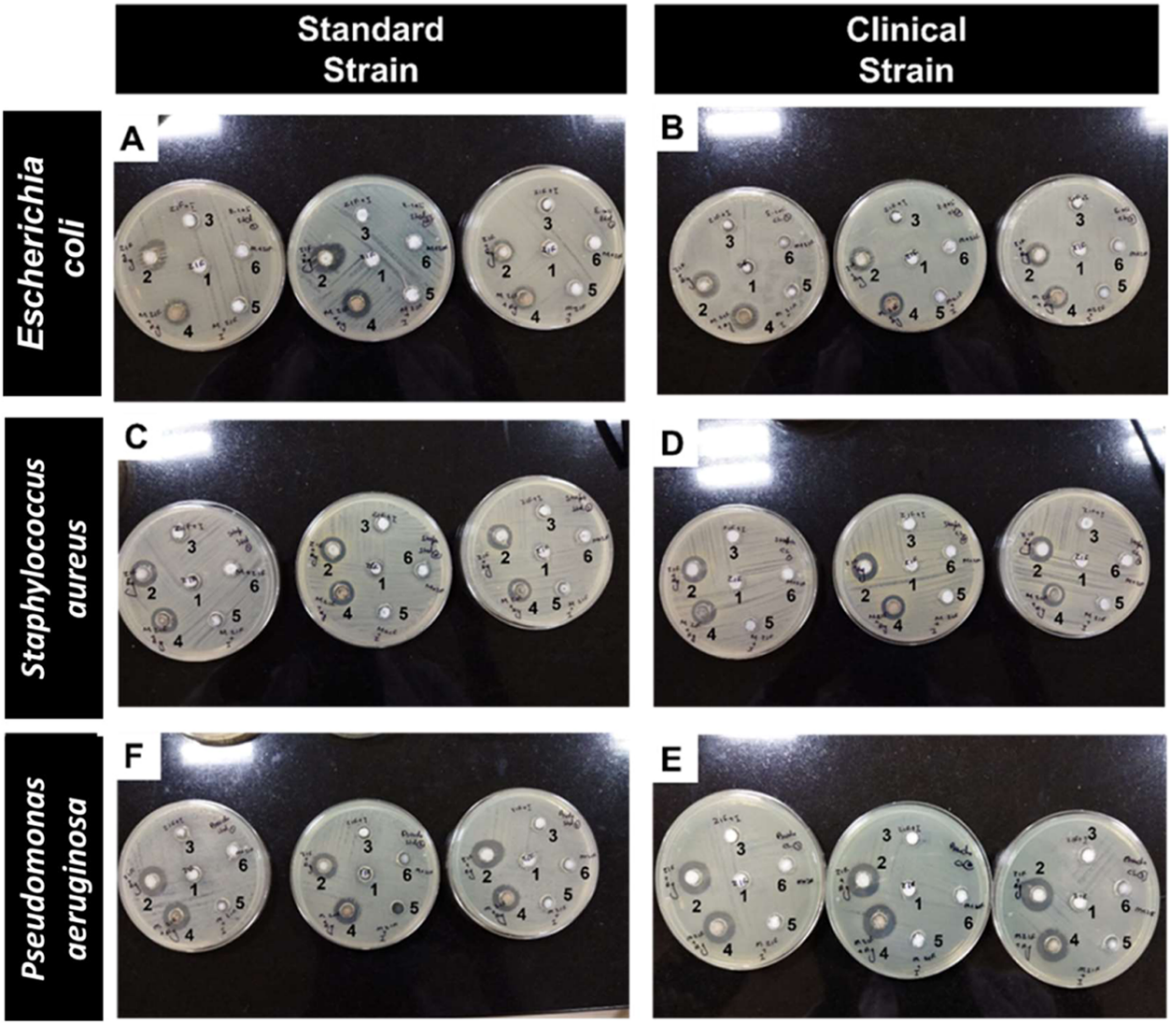
Antimicrobial properties of ZIF-L and Ag/ZIF-L. (A–E) Representative images of growth inhibition by nanoparticles on either standard or clinical strain of S. aureus, E. coli, or P. aeruginosa (1: ZIF-L (10 mg/mL), 2: Ag/ZIF-L (10 mg/mL), 3: ZIF-I, 4: mannose-coated Ag/ZIF-L, 5: mannose-coated ZIF-I, and 6: mannose-coated ZIF-L).

**Figure 7:**
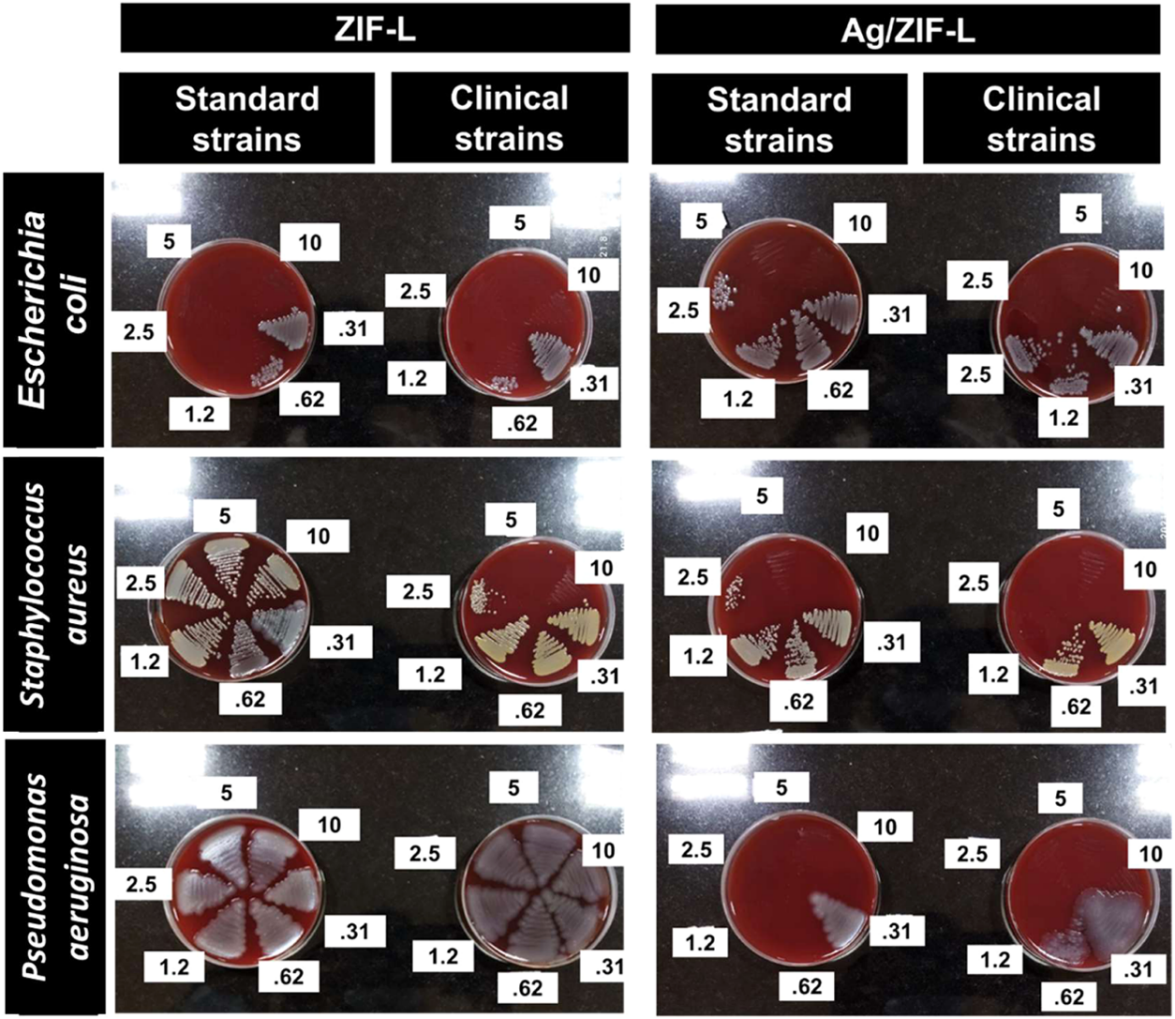
Minimum bactericidal concentration (MBC) studies of ZIF-L and Ag/ZIF-L nanoparticles with clinical and standard strains of S. aureus, E. coli, or P. aeruginosa (10, 5, 2.5, 1.25, 0.625, and 0.3 mg/mL). Each group of experiments was repeated thrice.

**Figure 8:**
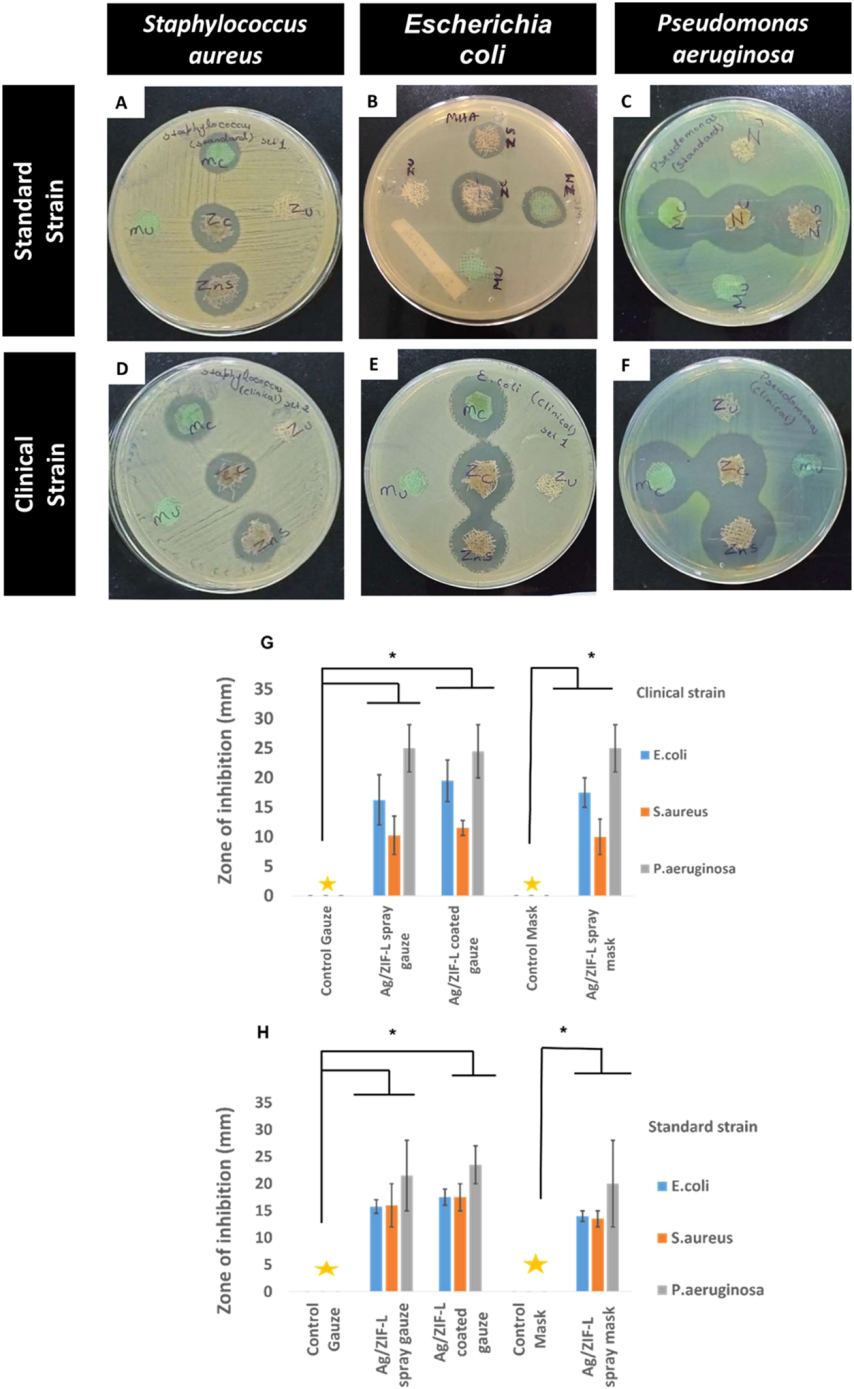
Antimicrobial effect of Ag/ZIF-L nanoparticles either in-situ-coated or sprayed on a surgical gauze or mask. (A–F) Representative images of growth inhibition by Ag/ZIF nanoparticles on either standard or clinical strain of S. aureus, E. coli, and P. aeruginosa (1: mask not sprayed with Ag/ZIF-L, 2: mask sprayed with Ag/ZIF-L, 3: in situ coating of gauze with Ag/ZIF-L, 4: gauze sprayed with Ag/ZIF-L, and 5: gauze without coating with Ag/ZIF-L). (G) Measured zone of inhibition on standard strains. (H) Measured zone of inhibition on clinical strains. Data are represented as mean ± SEM of three independent experiments. *p < 0.053.

**Table 3:** Bactericidal Activity of Silver-Based MOFs Reported in the Literature

	ZOI for different bacterial strains (mm)			
MOF	E. coli	S. aureus	P. aeruginosa	ref
γ-Fe_2_O_3_@SiO_2_@ZIF-8-Ag (FSZ-Ag)	26.4	25.6		^62^
Ag@ZIF-8	10.8	14.8		^50^
Ag-NaA zeolite	16.3	14. 5		^63^
Ag/UiO-66-NH_^2^_	7.453	9.013		^64^
[Ag_2_(O-IPA)(H_2_O)·(H_3_O)]	20	16		^65^
[Ag_5_(PYDC)_2_(OH)]	17	14		
Ag/ZIF-L	22	20	24	our work

**Table 4:** Minimum Bactericidal Concentration (MBC) Studies of ZIF-L and Ag/ZIF-L Nanoparticles^a^

	E. coli		S. aureus		P. aeruginosa	
nanoparticles	standard strain	clinical strain	standard strain	clinical strain	standard strain	clinical strain
ZIF-L	0.625	1.25	>10	2.5	>10	>10
Ag/ZIF-L	2.5	1.25	2.5	0.625	0.3	0.625

a Concentrations in mg/mL.

### 3.3 Dye Absorption and Degradation

#### 3.3.1 Methylene Blue Dye Removal

Methylene blue dye was used to examine the elimination of dye from solution through the utilization of synthesized ZIF-L and Ag/ZIF-L nanoparticles. The dye solution containing nanoparticles was exposed to intense sunlight to determine the photocatalytic aspects of the nanoparticles. For comparative purposes, a parallel setup involving the dye–nanoparticle mixture was maintained under dark conditions, and the process of dye removal from the solution was documented. Figure 9 shows the UV–vis absorption studies for methylene blue dye, and Figure 10A,B shows the degradation at 10 μM concentration. We observed that exposing the dye to light caused faster dye removal for all groups exposed to light. ZIF-L showed slightly faster dye removal than the Ag/ZIF-L group (Figure 10A,B). A similar trend was also observed with higher concentrations of methylene blue dye at 100 and 1000 μM under light conditions, as presented in Figure S2 (Supporting Information), and methylene blue dye removal percentage at different concentrations (10, 100, and 1000 μM) under light conditions are summarized in Table 5. This could be due to the better adsorption of dye on the surface of ZIF-L due to or differences in the pore volume of ZIF-L and Ag/ZIF-L. Silver ions may be occupying the pores, resulting in lower adsorption of methylene blue dye and subsequent removal of the dye from the solution in the Ag/ZIF-L group. Around 150 min, we found that 100% of the dye was removed from the solution for both ZIF-L and Ag/ZIF-L nanoparticle groups exposed to sunlight. In both groups, methylene blue dye exposed to sunlight showed 100% dye removal at 180 min. In comparison, the ZIF-L group in the dark showed 83% dye removal, and Ag/ZIF-L showed 65% dye removal at 180 min, showing a similar rate of removal to the light-exposed group. The higher dye removal in the light-exposed group compared to the dark group could be explained by the photocatalytic activity on exposure to sunlight by ZIF-L and Ag/ZIF-L nanoparticles. The ZIF-L and Ag/ZIF-L nanoparticles were further characterized after the dye removal experiment using FTIR analysis. The FTIR peaks of ZIF-L and Ag/ZIF-L before and after dye removal under light and dark conditions showed no changes, suggesting that there is no degradation of the nanoparticles with or without the presence of sunlight during MB dye removal. The FTIR graph is illustrated in Figure S3 (Supporting Information). Our studies show that the incorporation of silver does not overtly change the rate of dye removal in comparison to ZIF-L. This may suggest that the primary means of dye removal was through dye adsorption rather than catalytic degradation, which is represented by ZIF-L having better adsorption than Ag/ZIF-L under dark conditions. Methylene blue is a cationic dye, while ZIF-L is a positively charged nanoparticle, and the doping of other positively charged silver nanoparticles may hinder the adsorption capacity due to charge repulsion.

**Figure 9:**
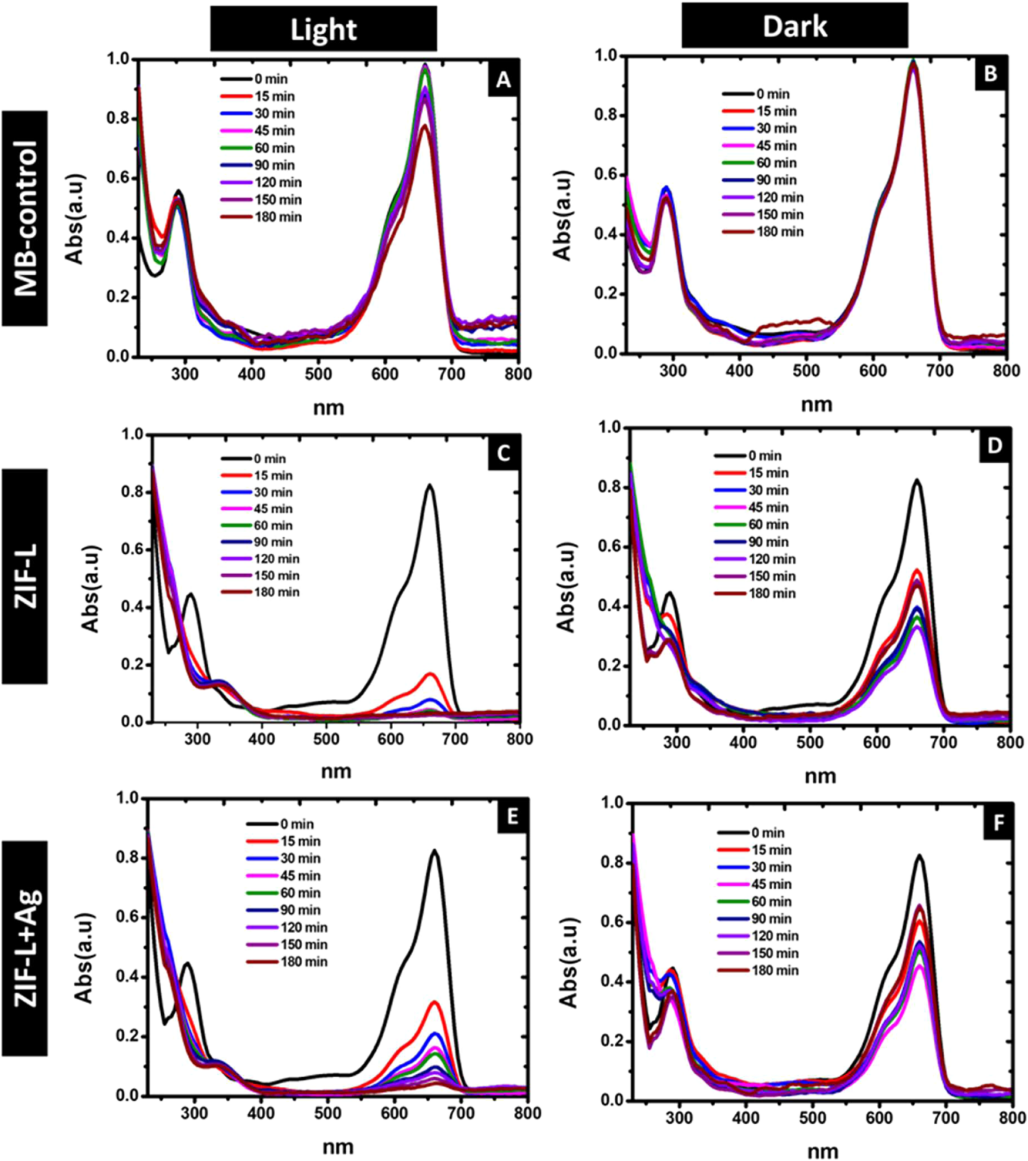
UV–vis spectroscopic studies of the degradation of methylene blue dye in the presence of ZIF-L and Ag/ZIF-L exposed to light and kept under dark conditions. (A) Control dye sample exposed to light, (B) control dye sample kept under dark conditions, (C) dye incubated with ZIF-L exposed to light, (D) dye incubated with ZIF-L kept under dark conditions, (E) dye incubated with Ag/ZIF-L exposed to light, and (F) dye incubated with ZIF-L kept under dark conditions.

**Figure 10:**
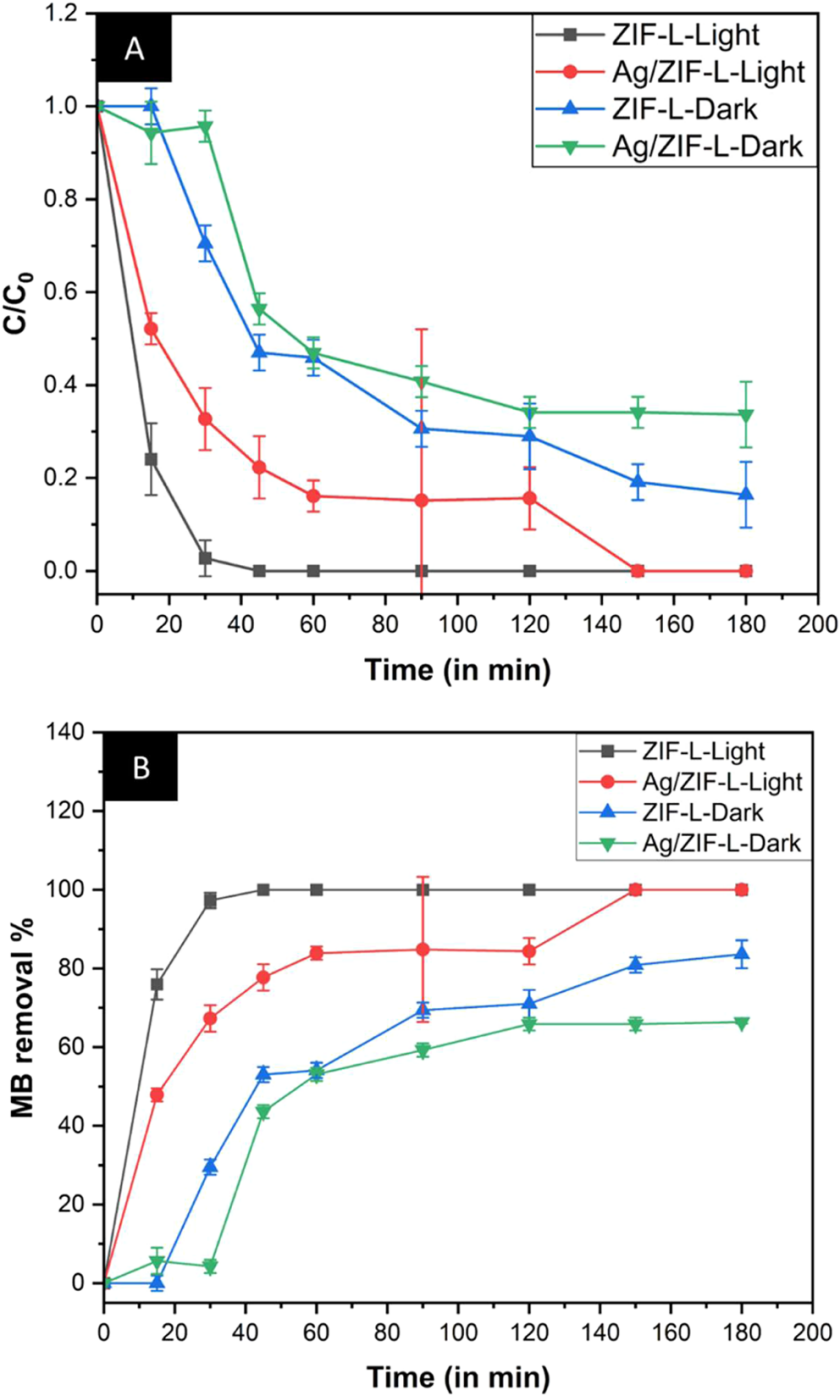
Removal of methylene blue dye from solution in the presence of ZIF-L and Ag/ZIF-L nanoparticles. Plots of (A) C/C_0_ versus time and (B) percentage of methylene blue dye removal versus time.

**Table 5:** Dye Removal of Different Concentrations of Methylene Blue under Light Conditions with ZIF-L and Ag/ZIF-L Nanoparticles

	dye removal % under light conditions after 3 h		
	10 μM MB	100 μM MB	1000 μM MB
ZIF-L	94.1 ± 3.66	45.45 ± 1.10	31.18 ± 1.15
Ag/ZIF-L	100 ± 0	38.32 ± 1.69	28.93 ± 0.35

#### 3.3.2 Cyclic Removal of Methylene Blue Dye

Determination of the continuous removal of dye was performed by cyclic degradation of methylene blue with ZIF-L and Ag/ZIF-L under light and dark conditions, and the dye removal percentage was determined after 3 h of each cycle. 10 and 100 μM dye concentrations were used to determine the dye removal percentage, which showed that at 10 μM dye concentration both ZIF-L and Ag/ZIF-L caused 100 and 95% of dye removal, respectively, after 3 h of exposure to sunlight for 5 continuous cycles, whereas under dark conditions, on average, 73 and 55% of dye removal were observed with ZIF-L and Ag/ZIF-L, respectively. A similar trend was observed with 100 μM methylene blue dye removal, where average values of 50 and 45% dye removal were observed with 5 cycles under light conditions and 12 and 8% dye removal were seen under dark conditions for ZIF-L and Ag/ZIF-L, respectively. Figure 11 illustrates the cyclic dye removal of methylene blue with ZIF-L and Ag/ZIF/L under light and dark conditions, respectively.

**Figure 11:**
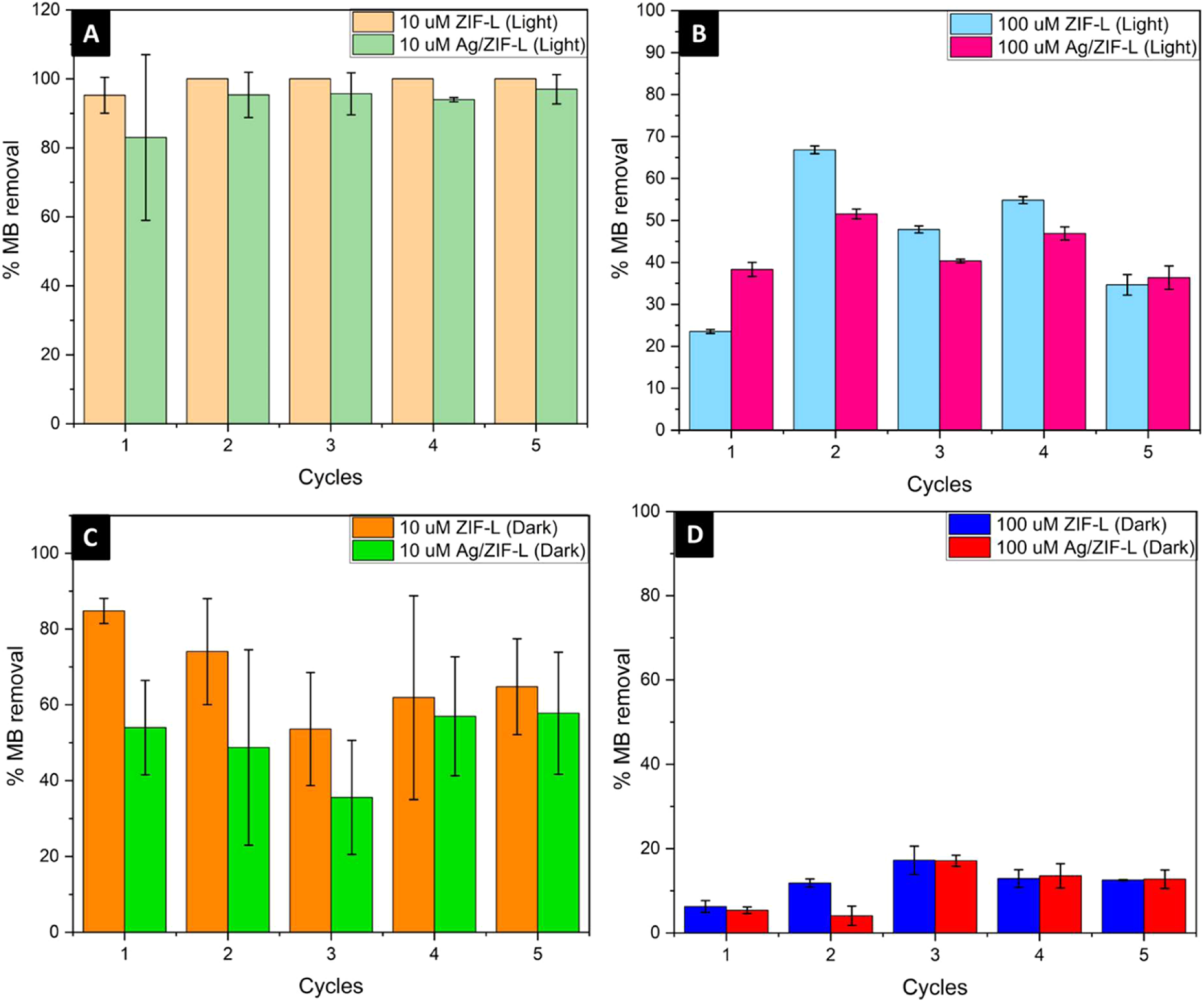
Cyclic degradation of different concentrations of methylene blue dye under light and dark conditions with ZIF-L and Ag/ZIF-L nanoparticles. (A) 10 μM MB under light conditions; (B) 100 μM MB under light conditions; (C) 10 μM MB under dark conditions; (D) 100 μM MB under dark conditions.

#### 3.3.3 Congo Red (Azo Dye) Dye Removal

To further understand the photocatalytic potential of Ag/ZIF-L nanoparticles, another class of dyes such as Congo red (anionic dye) was used for dye removal studies. The dye removal percentages of Congo red with ZIF-L and Ag/ZIF-L under light and dark conditions are summarized in Table 6. UV–vis spectroscopic studies of the degradation of Congo red dye in the presence of ZIF-L and Ag/ZIF-L when exposed to light and kept under dark conditions are illustrated in Figure S4 (Supporting Information).

**Table 6:** Dye Removal Percentage of Congo Red Dye with ZIF-L and Ag/ZIF-L under Light and Dark Conditions

	congo red dye removal % under light and dark conditions after 3 h		
	concentration (μM)	light	dark
ZIF-L	250	100 ± 0	100 ± 0
	500	94 ± 1.6	90 ± 0.6
	1000	94 ± 0.5	84 ± 0.4
Ag/ZIF- L	250	100 ± 0	100 ± 0
	500	97 ± 1.4	91 ± 1.7
	1000	94 ± 0.6	88 ± 1.1

In conclusion, incorporating silver also did not hamper the dye removal process. Further, from antimicrobial studies, the incorporation of silver is beneficial for killing bacteria in polluted waste.

### 3.4 Methylene Blue Degradation and Byproduct Analysis

The degradation byproducts of methylene blue upon reaction with ZIF-L and Ag/ZIF-L under light and dark conditions were analyzed using LC–MS. The chromatogram peak of methylene blue (control) was observed at a retention time (RT) of 1.89 (Figure S5A, Supporting Information). The full mass spectrum of methylene blue illustrated in Figure S6 (Supporting Information) shows a peak at *m*/*z* 284.23, confirming the presence of methylene blue. The reaction of ZIF-L and Ag/ZIF-L with methylene blue under light and dark conditions was performed, and the chromatogram was obtained. Under dark conditions, both ZIF-L and Ag/ZIF-L yielded 3 different chromatogram peaks at RT 2.14, 1.60, and 1.40, which are shown in Figure S5B,C (Supporting Information), whereas under light conditions only 2 major chromatogram peaks were observed at RT 2.14 and 1.40, which are shown in Figure S5D,E (Supporting Information). The full mass spectrum of the chromatograms showed an RT peak at 2.14 having an *m*/*z* value of 284 compared to the control methylene blue whose RT peak was at 1.89. The demethylated byproduct such as azure B (AB) having an *m*/*z* value of 270 was observed for both ZIF-L and Ag/ZIF-L under dark and light conditions, as illustrated in Figures S7A–S10A (Supporting Information), respectively. A shift in the RT compared to the control methylene blue (RT = 1.89) was observed.

The other degraded methylene blue byproducts were observed at RT 1.60 and 1.40 under dark conditions, which are presented in Figure S7B,C and S8B,C, and only one RT peak at 1.40 was observed under light conditions in both ZIF-L and Ag/ZIF-L groups, which are shown in Figure S9B and S10C. The mass spectral analysis of the peaks at RT 1.60 and 1.40 showed that the *m*/*z* value of 284 corresponding to methylene blue was absent, and the subsequent peaks of the degraded byproducts were observed at low *m*/*z* values. The major degradation products of methylene blue are demethylation derivatives like azure A (*m*/*z* 256), azure B (*m*/*z* 270), azure C (*m*/*z* 242), and thionin (*m*/*z* 228), as reported in the literature.^66,67^ Our results showed that the major peaks from methylene blue degradation were observed at RT 1.40 and 1.60 RT, which correspond to hydroxylated, deaminated, and ring-cleavage byproducts. In comparison, the demethylated degradation products were less compared to the hydroxylated, deaminated, and ring-cleavage byproducts. This suggests that extensive degradation after complete demethylation occurred leading to ring opening of methylene blue.^66,68–70^ The degradation pathway and the structures of major degradation products and fragment byproducts corresponding to the mass spectra are shown in Figure 12. The degradation products from A1 to A5 represent the demethylation pathway of methylene blue, B1 and B2 are hydroxylated byproducts, and B3 to B5 are deaminated and ring-cleavage byproducts of methylene blue.

**Figure 12:**
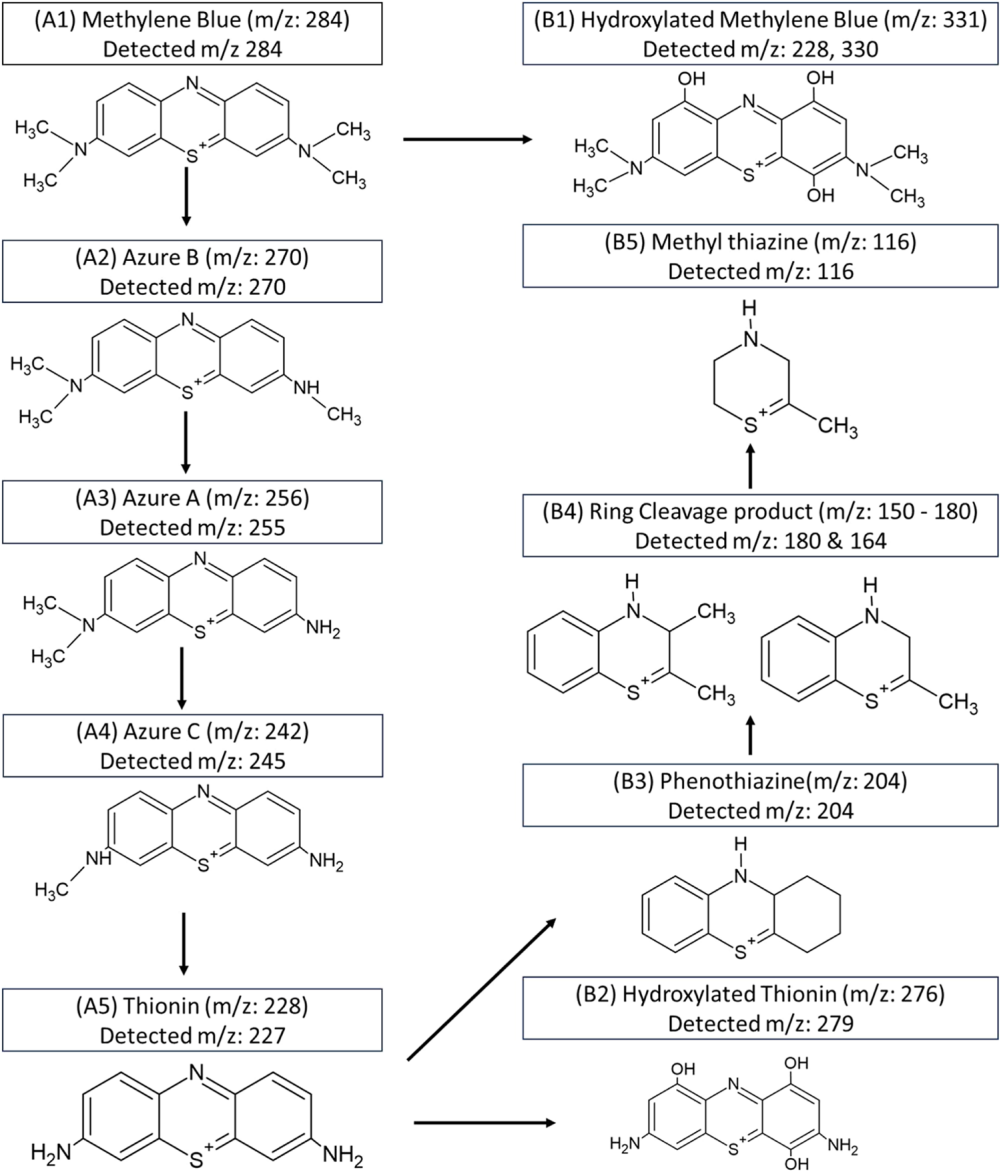
Degradation pathway of methylene blue representing major degraded products and fragmentation byproducts.

In this study, we demonstrated the potential antimicrobial activity and dye degradation ability of ZIF-L and Ag/ZIF-L nanoparticles. While previous studies have highlighted the antimicrobial properties of ZIF-L nanoparticles, our study did not show significant activity against S. aureus and P. aeruginosa strains. The incorporation of silver (Ag/ZIF-L) enhanced the activity of ZIF-L against all of the strains tested. We also incorporated iodine (ZIF-I) and tried coating the nanoparticles with mannose to check its bactericidal activity, as it has been cited in the literature that coating of mannose with polymers and other particles have shown increased bactericidal activity.^71,72^ The rationale of the study was to improve the antibacterial activity for ZIF-L coated with mannose. However, this experiment did not show many marked increases in the antimicrobial activity compared with the incorporation of silver. The coating of Ag/ZIF-L nanoparticles on the gauze and mask demonstrated the ability of these nanoparticles to be either spray-coated or in-situ-coated on various surfaces. SEM imaging and EDS spectroscopy of the coated gauze or mask confirmed the coating of these nanoparticles. These results show the suitability of these nanoparticles to be coated on a wide variety of surfaces. We further tested the ability of these nanoparticles to remove the organic dye. In our study, we used methylene blue as a model dye and studied the degradation of the dye under dark and exposure to sunlight. We observed an increase in dye removal under light conditions, with ZIF-L showing better removal of the dye compared to Ag/ZIF-L. However, incorporation of silver (Ag/ZIF-L) did not hamper the dye removal ability, and we observed complete dye removal. LC–MS data showed three major retention times for ZIF-L and Ag/ZIF-L under dark conditions versus two prominent peaks under light conditions for ZIF-L and Ag/ZIF-L groups. This suggests that under light conditions the degradation mechanism could be different from that when not exposed to light. The primary mechanism of dye removal could be adsorption of the dye, and under light conditions additional degradation could occur. We observed higher degradation for ZIF-L in comparison to the Ag/ZIF-L group. The LC–MS data correlate well with the spectrophotometry study for dye degradation, where it was observed that ZIF-L showed faster removal of the dye compared to Ag/ZIF-L. One possible reason could be due to cationic silver present in the ZIF MOF, which may hinder the adsorption of dye on the surface of Ag/ZIF-L.

The potential application of Ag/ZIF-L could be in environmental remediation. These nanoparticles can be coated on various surfaces like gauze, which further can be made into sustainable filters that can be used for remediation of biological and organic pollutants. The study performed by Reddy et al. used polymer-coated gauze as a sustainable and economical absorbent material for removal of nickel ions.^73^ In another study, Chang et al. used a polymer/BiOBr-modified gauze for removal of heavy metals and photocatalytic decoloration of dyes.^74^ Further researchers have used a gauze-coated biopolymer for antimicrobial water filters and for removal of metal ions.^75–77^ These studies show the potential of Ag/ZIF-L for environmental remediation application.

## 4 Conclusions

In this study, ZIF-L and Ag/ZIF-L nanoparticles were synthesized by the precipitation method and doped with silver to derive the nanoformulation. The nanoparticles were characterized using FTIR, XRD, and SEM analyses, and the presence of zinc and silver ions was confirmed by EDS and ICP–OES studies. The ZIF-L and Ag/ZIF-L nanoparticles were found to be thermally stable. The zinc and silver concentrations within the nanoparticles were around 16 and 1.9 μg/mg nanoparticles. The antimicrobial properties of ZIF-L and Ag/ZIF-L nanoparticles were tested on standard and clinical isolates of S. aureus, E. coli, and P. aeruginosa. The antimicrobial activity of ZIF-L nanoparticles was observed only against E. coli and S. aureus strains at higher concentrations. In comparison, Ag/ZIF-L showed better activity against all strains of bacteria. The synthesized nanoparticles were coated on various surfaces, and the antimicrobial properties of the coated surface were demonstrated. Further, the dye removal properties of the synthesized nanoparticles were also studied. These studies demonstrate the beneficial effect of Ag/ZIF-L nanoparticles for healthcare and environmental remediation applications. ZIF-L and Ag/ZIF-L nanoparticles effectively removed methylene blue dye from solution, whose photocatalytic activity enhanced with light exposure. While silver incorporation did not increase dye removal rates, it has added beneficial antimicrobial properties, making Ag/ZIF-L promising for treating dye and bacterial pollutants in wastewater. Further, both ZIF-L and Ag/ZIF-L showed similar dye degradation byproducts under light and dark conditions, which was confirmed using LC–MS data.

## Supplementary Material



## Data Availability

All data for this communication article were created by M.P., using the facilities in the Department of Biomedical Engineering, Manipal Institute of Technology, MAHE. The data supporting this article are included in the main text as well as in the Supporting Information.

## Notes

Preparation of iodine-doped and mannose-coated ZIF-L-doped nanoparticles; preparation technique for the coating of mask and gauze with ZIF-L and Ag/ZIF-L nanoparticles for antimicrobial studies; characterization of nanoparticle coating of mask and gauze by SEM–EDS, FTIR, and UV–vis analyses; dye degradation of methylene blue and Congo red with ZIF-L and Ag/ZIF-L nanoparticles under light and dark conditions; and LC–MS byproduct analysis of methylene blue dye after the reaction (PDF)

## #

V.B.R. died prior to the publication of this work.
