# Supplementary material for: Evaluation of the Antimicrobial and Organic Dye Removal Properties of Silver-Incorporated ZIF‑L Metal–Organic Framework Nanoparticles
Source: ACS Omega. 2025 May 23;10(21):21334–49. doi: 10.1021/acsomega.4c11412 (PMC12138686; doi:10.1021/acsomega.4c11412)
Supplement: Supplementary file 1 [file ao4c11412_si_001.pdf]

Evaluation of the antimicrobial and organic dye removal properties of silver-incorporated ZIF-L Metal-Organic Framework (MOF) nanoparticles.

Sreejith Govindan[1†], Chaithra S[1,2†], Rudra Nath Ghosh[3†], Vaidehi B R[3‡], Ashutosh Gupta[4], Sudheer Moorkoth[4], Pramod K Namboothiri[3], Mathew Peter[3*]

[1] *Department of Basic Medical Sciences, Manipal, Manipal Academy of Higher Education, Manipal*

[2] *Department of Emergency Medicine, Kasturba Medical College, Manipal Academy of Higher Education, Manipal*

[3] *Department of Biomedical Engineering, Manipal Institute of Technology, Manipal, Manipal Academy of Higher Education, Manipal*

[4] *Department of Pharmaceutical Quality Assurance, Manipal College of Pharmaceutical Sciences, Manipal Academy of Higher Education, Manipal*

† Author's equal contribution

*Corresponding author:

Address: Department of Biomedical Engineering, Manipal Institute of Technology, Manipal, Manipal Academy of Higher Education, Manipal

E-mail: mathew.peter@manipal.edu

Supplementary Information

1. Preparation of ZIF-I nanoparticles

0.04 M Zinc nitrate solution with Potassium iodine (0.022 M) was reacted with 0.03 M 2-methylimidazole solution, and the mixture was kept under constant stirring for 24 hours at room temperature in dark conditions. The precipitate was then allowed to settle down, filtered and collected. The product is then washed with 80% methanol and dried.

2. Preparation of Mannose-coated ZIF-L, Ag/ZIF-L and ZIF-I nanoparticles

Mannose coating nanoparticles (ZIF-L, Ag/ZIF-L or ZIF-I) were prepared by incubating the nanoparticles with 6 mg/ml mannose solution and stirred continuously for 3 hours. The solution was centrifuged and the precipitate was collected and washed with 80% methanol and dried.

3. Metal-Organic Framework coating process

3.1. Spray coating

Spray coating of gauze and mask was done using prepared nanoparticles of ZIF-L and Ag/ZIF-L as described in the main text. For coating 10 mg/ml of nanoparticles were dissolved in methanol solution. The nanoparticle suspension was transferred to a spray bottle and sprayed evenly on the surface of the gauze or mask. To maintain uniformity the surface was sprayed three times with the nanoparticle suspension and air dried. The coated gauze or mask was used in the antibacterial studies.

3.2. In-situ coating

In-situ coating of gauze and mask was done by incubating in Zinc nitrate solution followed by incubation in 2-methyl imidazolate solution for 30 min duration. The procedure was repeated for three cycles. For Ag/ZIF-L the silver nitrate was dissolved along with Zinc nitrate solution and incubated as per the procedure mentioned above.

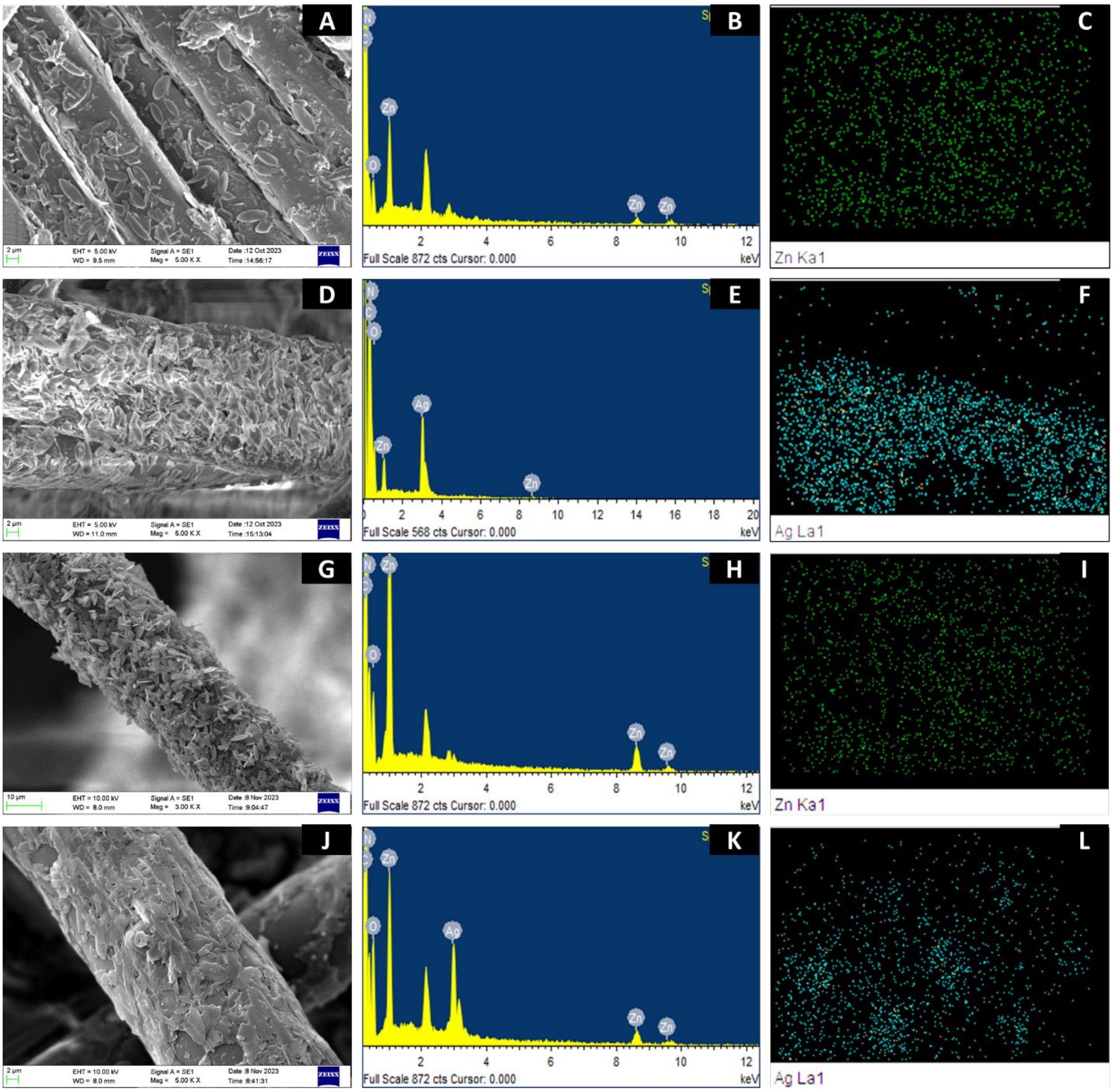

Figure S1: Scanning electron microscopy of (A) ZIF-L nanoparticles coated on cotton gauze, (D) Ag/ZIF-L nanoparticles coated on cotton gauze, (G) ZIF-L nanoparticles coated on mask and (J) Ag/ZIF-L nanoparticles coated on mask. Energy dispersive spectrum (EDS) of (B) ZIF-L nanoparticles coated on cotton gauze, (E) Ag/ZIF-L nanoparticles coated on cotton gauze, (H) ZIF-L nanoparticles coated on mask and (K) Ag/ZIF-L nanoparticles coated on mask. Energy dispersive spectrum map of (C, F) Ag/ZIF-L nanoparticles coated on cotton gauze and (I, L) Ag/ZIF-L nanoparticles coated on mask.

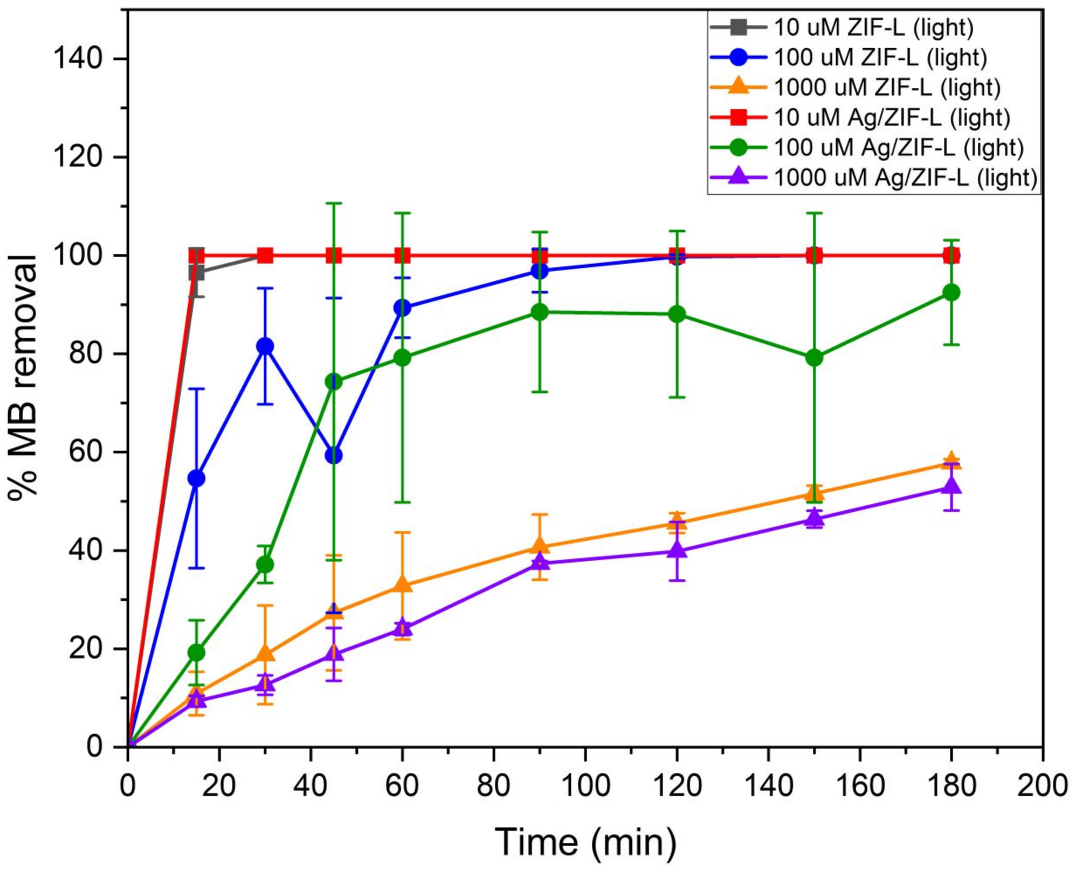

Figure S2: Different concentration dye removal percentage of Methylene blue dye with ZIF-L and Ag/ZIF-L under light condition.

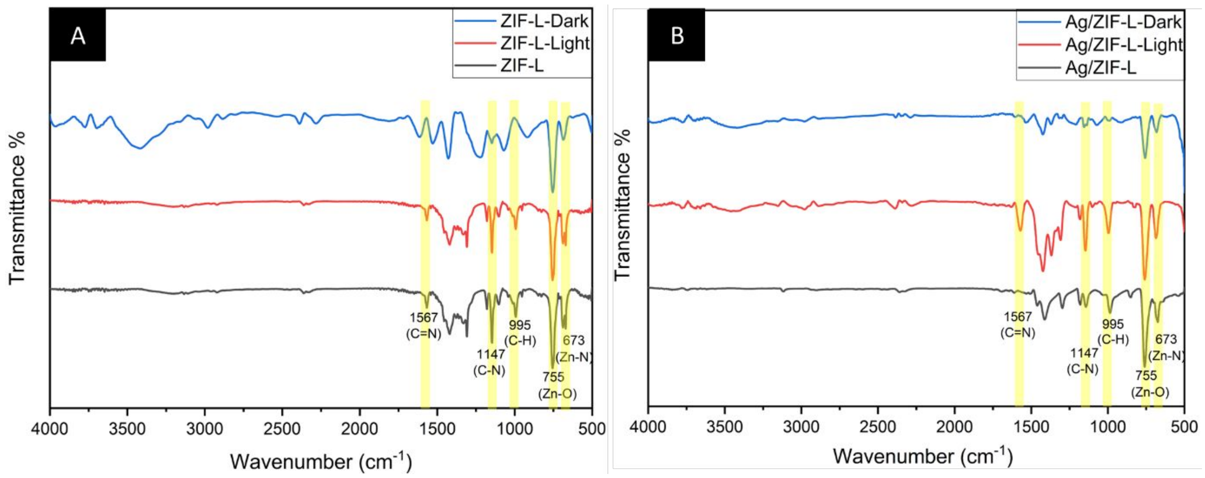

Figure S3: FTIR spectra of nanoparticles before and after dye removal under light and dark condition. (A) ZIF-L nanoparticles and (B) Ag/ZIF-L nanoparticles.

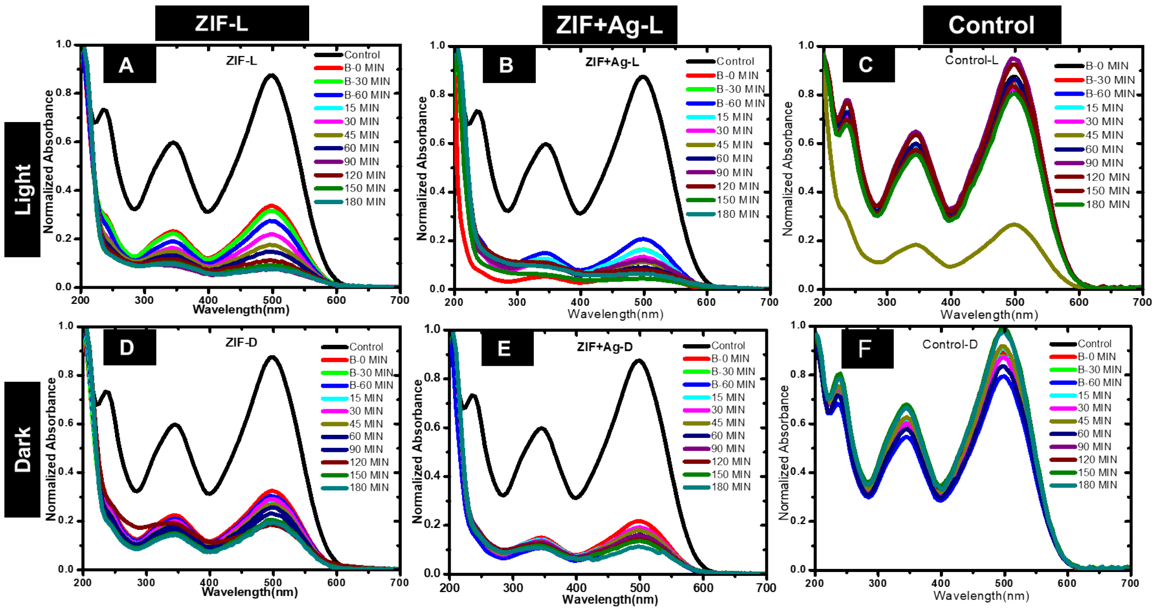

Figure S4: UV -VIS spectroscopic studies of the degradation of Congo Red dye in the presence of ZIF-L and Ag/ZIF-L exposed to light and kept under Dark conditions. (A) Dye incubated with ZIF-L exposed to light, (B) Dye incubated with Ag/ZIF-L exposed to light, (C) Control dye sample exposed to light, (D) Dye incubated with ZIF-L kept under dark conditions. (E) Dye incubated with Ag/ZIF-L kept under dark conditions and (F) Control dye sample kept under dark conditions.

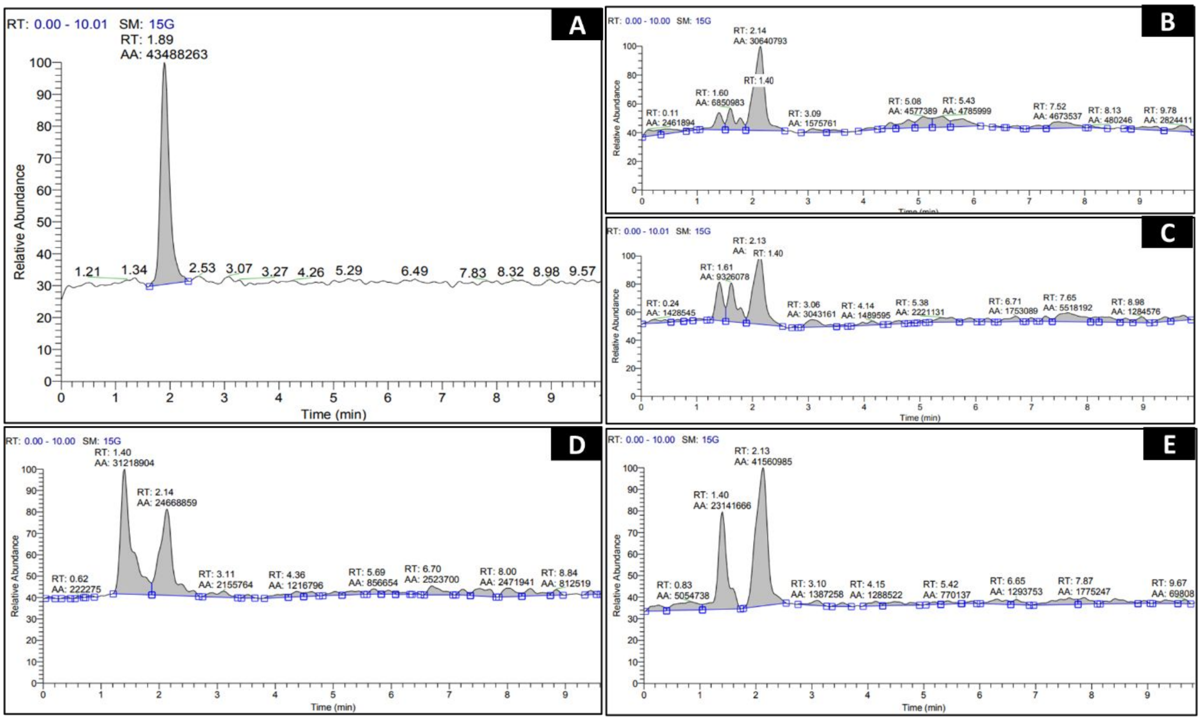

Figure S5: The chromatogram spectrum of (A) Methylene blue; (B) Methylene blue reaction with ZIF-L under dark condition; (C) Methylene blue reaction with Ag/ZIF-L under dark

condition; (D) Methylene blue reaction with ZIF-L under light and (E) Methylene blue reaction with Ag/ZIF-L under light.

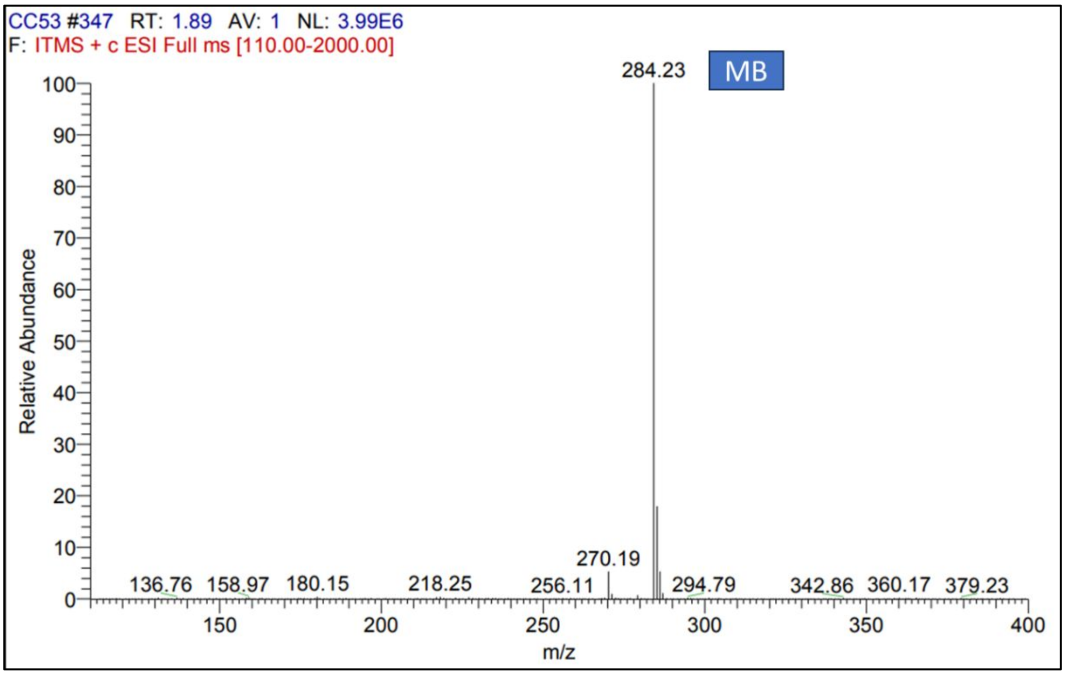

Figure S6: Mass Spectra of Methylene blue dye at RT 1.89.

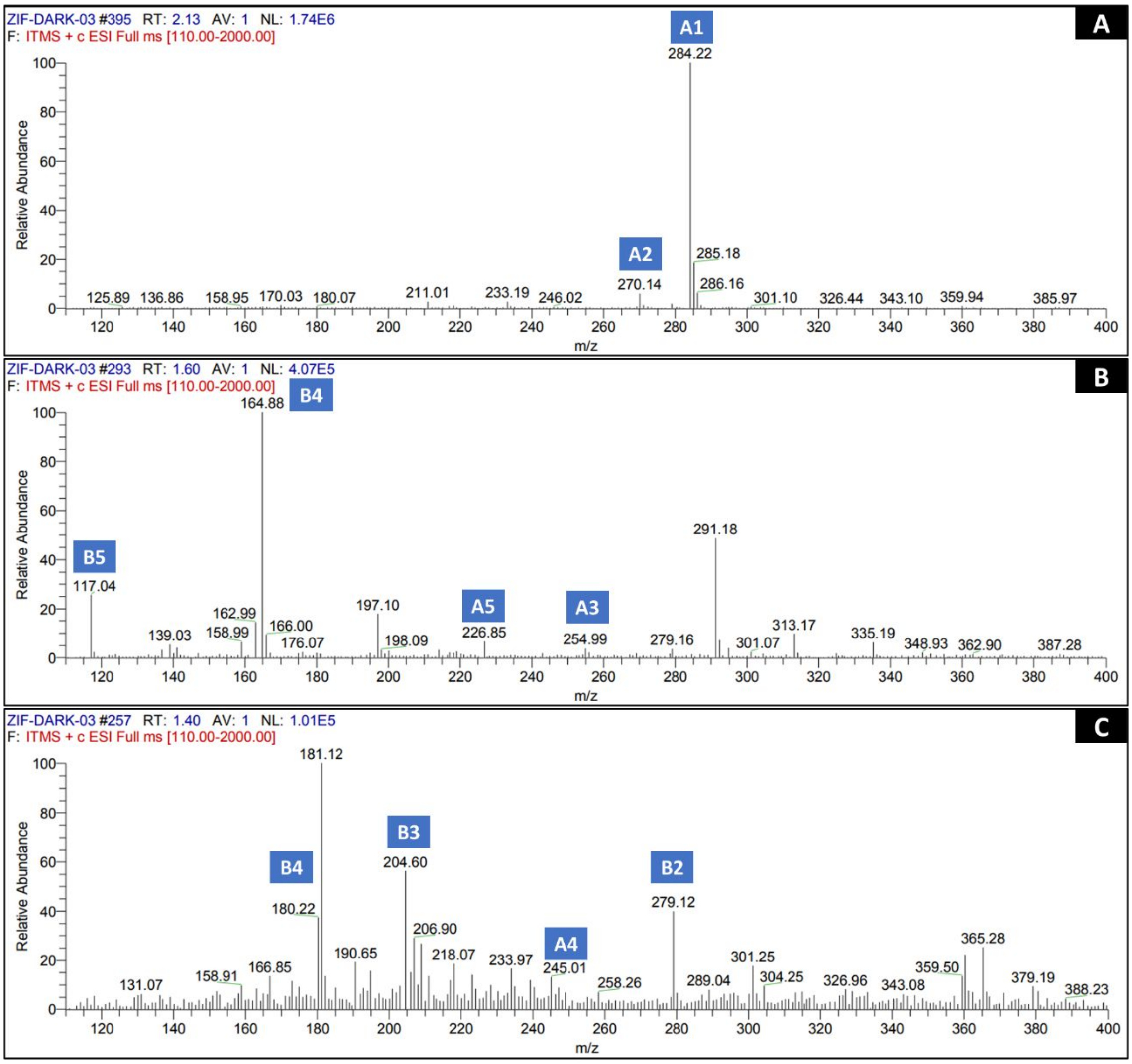

Figure S7: Mass spectra after Methylene blue degradation with ZIF-L under dark condition at (A) RT 2.13, (B) RT 1.60 and (C) RT 1.40.

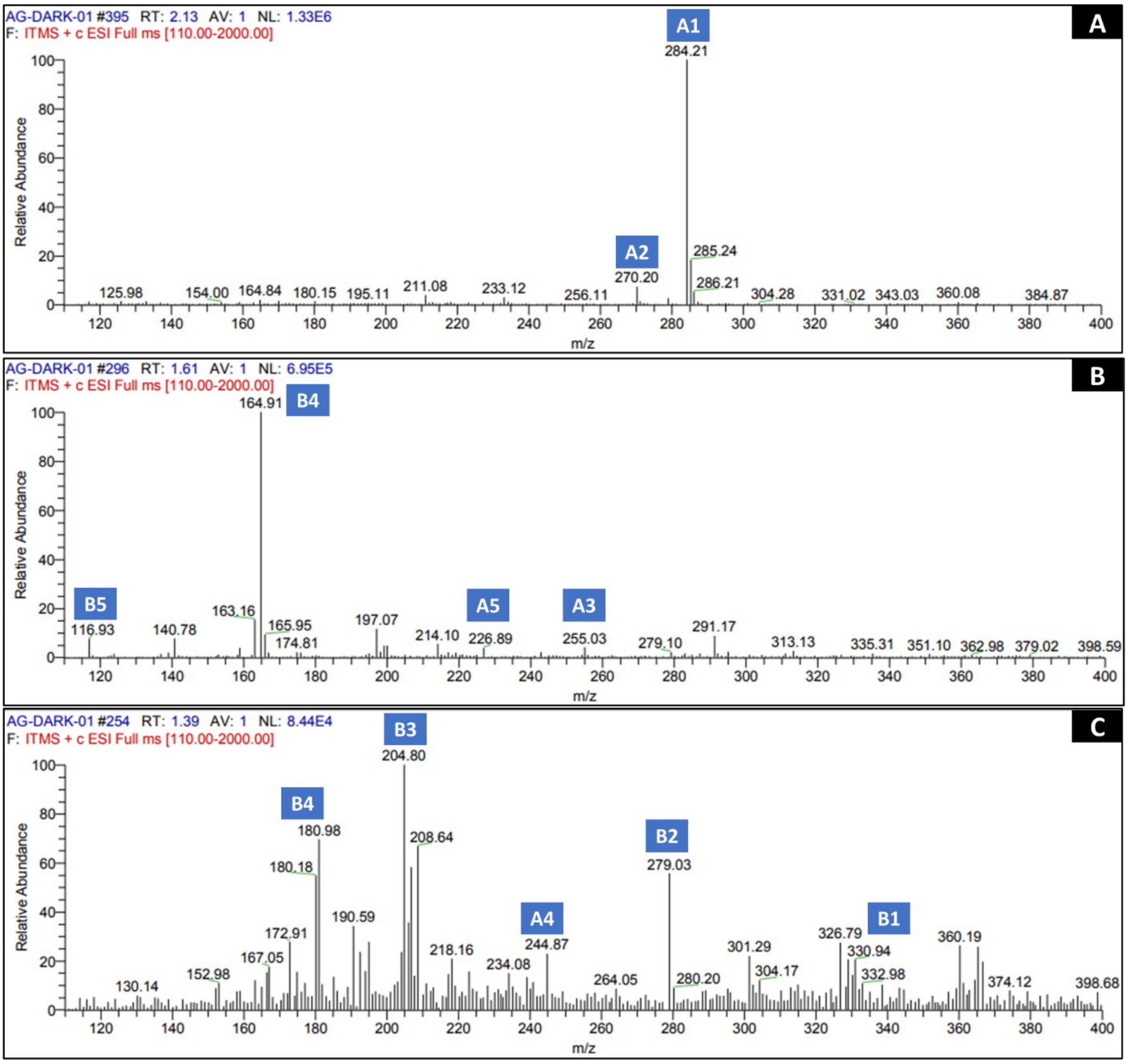

Figure S8: Mass spectra after Methylene blue degradation with Ag/ZIF-L under dark condition at (A) RT 2.13, (B) RT 1.61 and (C) RT 1.39.

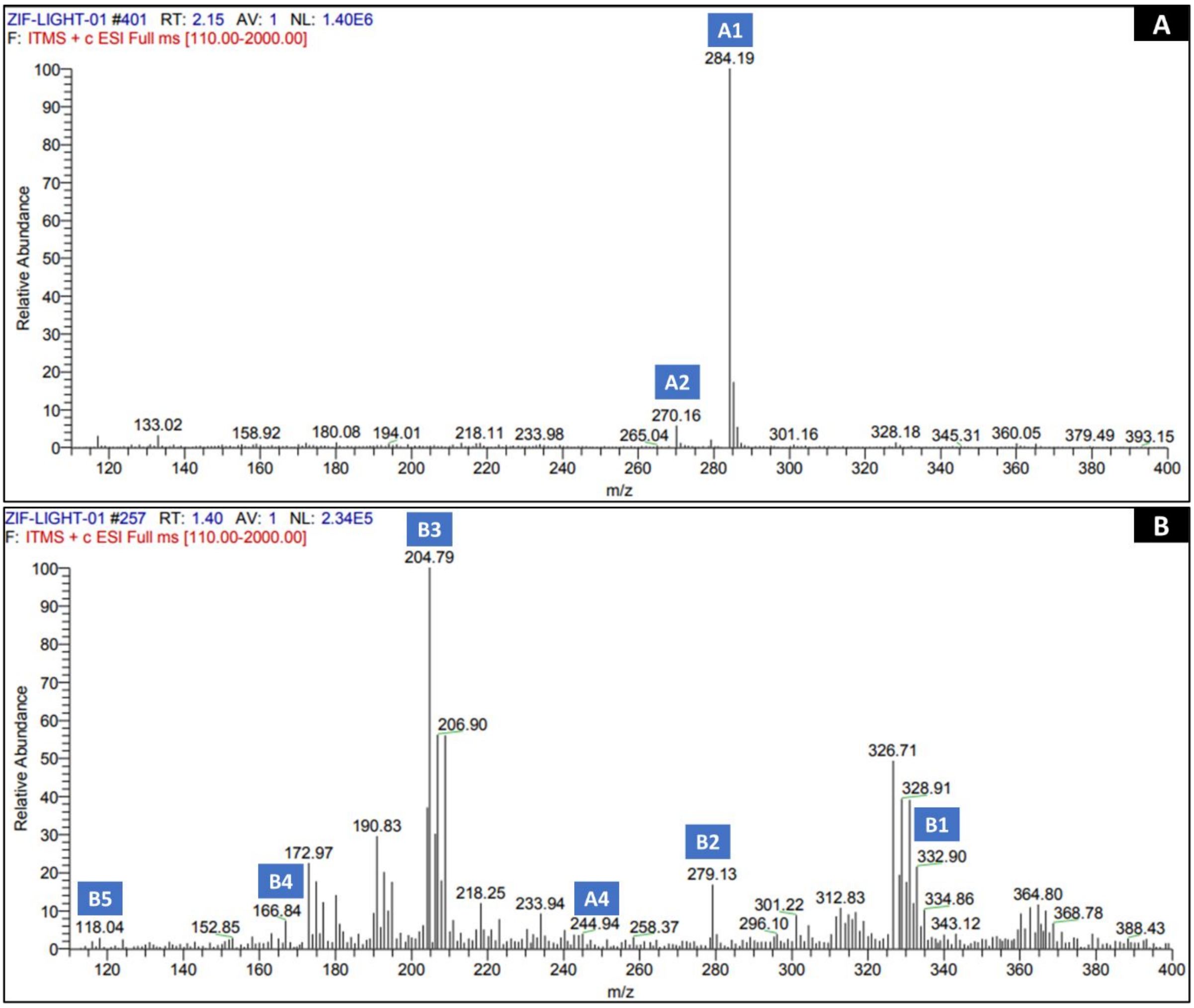

Figure S9: Mass spectra after Methylene blue degradation with ZIF-L under light condition at (A) RT 2.15 and (B) RT 1.40.

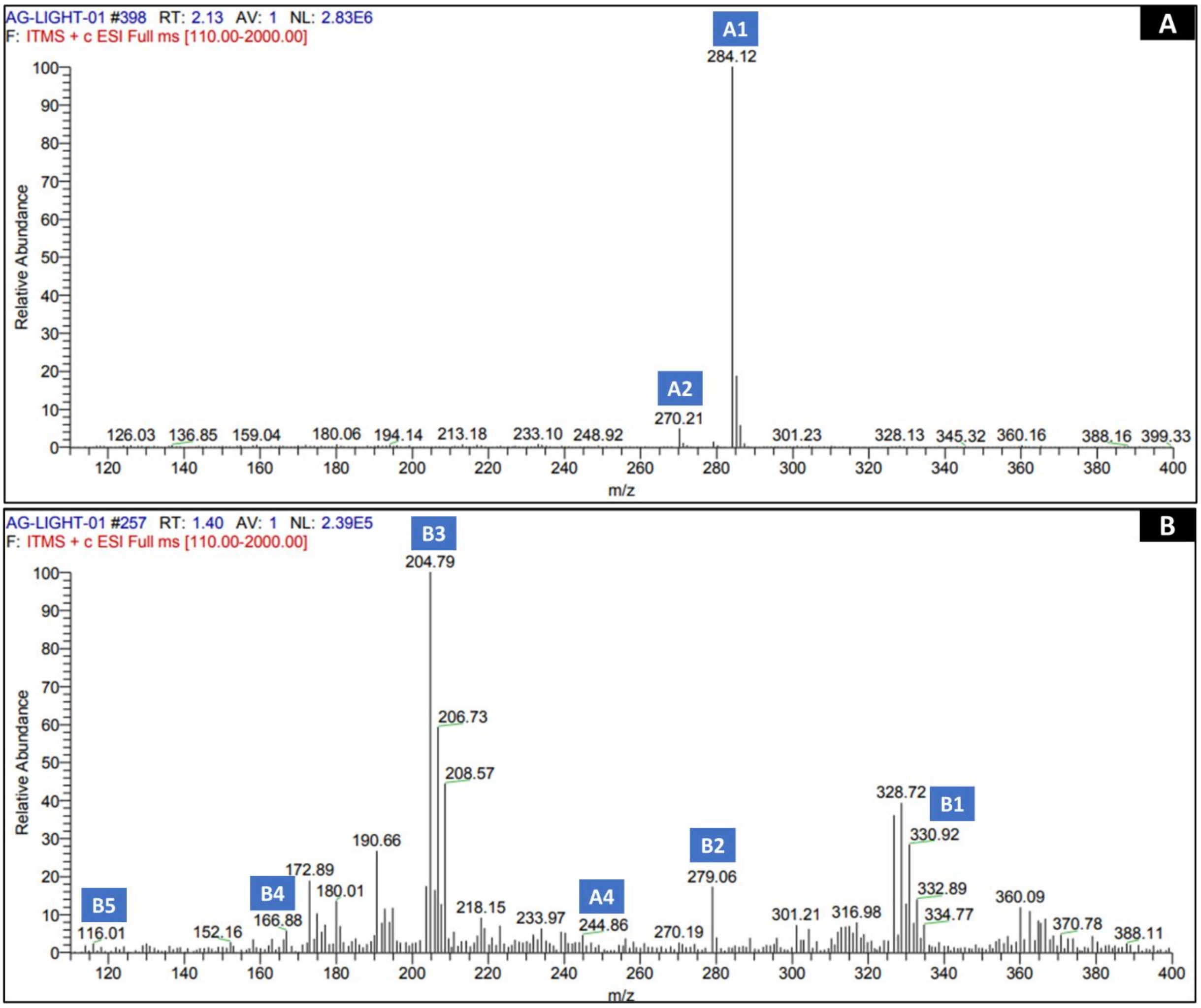

Figure S10: Mass spectra after Methylene blue degradation with Ag/ZIF-L under light condition at (A) RT 2.13 and (B) RT 1.40.
